# Retributive Philanthropy

**DOI:** 10.1177/00222437251320021

**Published:** 2025-02-06

**Authors:** Ethan Milne, Kirk Kristofferson, Miranda R. Goode

**Keywords:** prosocial behavior, donations, consumer aggression, retribution

## Abstract

Prosocial behavior research has historically considered altruistic or self-interested motives as the primary drivers for charitable giving. Recently, however, there have been many high-profile cases wherein consumers use their donations to harm others. The authors define this behavior, characterized by a desire for retribution resulting from witnessing or experiencing volitional wrongdoing, as “retributive philanthropy” and examine this phenomenon using a multimethod approach. Qualitative interviews with perpetrators and targets of retributive philanthropy reveal key themes of blameworthiness judgments, strong negative affect, and a desire to harm as a terminal goal of donation—none which are typically associated with prosocial behaviors. Analysis of real-world antivaccine protestor donation data finds similar themes of perceived wrongdoing and outrage related to retributive donations in a large-scale context. Five lab studies and five supplementary studies then demonstrate the effects of perceived volitional wrongdoing, harm, efficacy, and authoritarianism on willingness to make retributive donations. Together, these findings offer critical insight into an emerging mode of donation that is emotionally, motivationally, and behaviorally distinct from traditional prosocial behavior and has important implications for consumers and charitable marketers.

There's one person who has a special place in our hearts: Mike Pence. Today, break his heart and make a donation in his name.

—Planned Parenthood Action Fund (2019)

After the election of Donald Trump in 2016, organizations concerned with environmental advocacy, women's rights, and civil liberties received influxes of donations. Most notably, Planned Parenthood saw a 40-fold increase in donations in the months following the election (Preston 2017), an increase much larger than what other organizations experienced. What made Planned Parenthood different? It could be that donors believed abortion rights were uniquely threatened given the election of a conservative government. However, environmental protection and civil liberties were also at risk given the Trump platform yet did not realize the same increase. We propose that the influx of donations to Planned Parenthood may be attributed to a set of donation motives and behaviors that are distinct from those typically investigated by prosocial behavior researchers. After the election, consumers began donating to Planned Parenthood in Vice President Mike Pence's name and using his real address (Mettler 2016), resulting in the vice president receiving thousands of letters thanking him for supporting an organization he morally opposed. Upon recognizing this unique donation behavior, Planned Parenthood leveraged this phenomenon in its official advertisements, a strategic decision that led to over 82,000 consumers donating to the organization in Pence's name (Ryan 2016).

This style of donation has recently expanded to multiple causes. For example, after Russia's invasion of Ukraine in 2022, some consumers donated to a Ukrainian nongovernmental organization called “Sign My Rocket.” This organization gave consumers the opportunity to inscribe personalized retributive messages on artillery shells to be shot at Russian soldiers in exchange for a donation (Jankowicz 2022). Table 1 outlines further examples of this phenomenon. Importantly, these examples represent a diversity of political and moral viewpoints, and the punitive actions taken are provoked by situations representing a wide range of potential wrongdoing. Nevertheless, from the perspective of the donating consumers, all of these situations involve punishment of perceived wrongdoers through donation. These donations differ considerably from contemporary examples of prosocial behavior studied in extant marketing and consumer research and, we argue, constitute a novel form of charitable giving that we refer to as retributive philanthropy. Specifically, we define retributive philanthropy as charitable acts undertaken to punish a volitional wrongdoer. In our research, we seek to establish empirical support for this type of philanthropy as retribution, thus demonstrating the relevance of retribution to our theoretical understanding of prosocial behavior and generating practical insights for charitable organizations interested in leveraging retributive donations.

**Table 1.** Examples of Retributive Philanthropy.

**Retributive Donation Examples in Practice**	**Source**
In response to fatphobic statements from Abercrombie & Fitch's CEO, one consumer started a viral YouTube campaign that supported donations of A&F clothes to homeless people to harm the brand.	Glazek (2013)
The Toronto Zoo Wildlife Conservatory launched a Valentine's Day donation campaign. Consumers “nursing a broken heart” could donate $25 to name a cockroach after their ex-partner. A certificate was sent to their ex informing them that they had a cockroach namesake.	Djan (2023)
A Twitch streamer started a livestream that raised funds for Mermaids UK, a prominent transgender rights charity, to upset a transphobic comedy writer: “Well done … tons of people know about Mermaids and support them just to spite you!”	Griffin (2019)
In response to antichoice politician Rep. Matt Gaetz insulting a teenage activist for being overweight, the teen started a Planned Parenthood fundraiser with the hashtag “#MattGaetzIsProAbortion,” which has raised over $2 million.	Latifi (2022)
A journalist made donations to vaccine funds in the name of antivaccine protestors she argued with on Twitter.	Sommerland (2019)

Our mixed-methods approach includes qualitative interviews, analysis of real-world donation data, and lab experiments to investigate this distinct phenomenon. We demonstrate that retributive philanthropy requires perceptions of volitional wrongdoing, negative moral judgments, and a desire to punish. These findings sharply contrast with previous research suggesting that prosocial behavior is characterized by positive emotions like love (Cavanaugh, Bettman, and Luce 2015) or gratitude (Bartlett and DeSteno 2006), positive relationships (Sepehri et al. 2021), and wanting to help others (Batson 2022). Although recent research and popular press opinion pieces have begun to discuss how rage or spite might motivate donations (Taylor and Miller-Stevens 2022; Witkowsky 2021), these dialogues do not consider how donations might be used as a vehicle for punishment, and as such are unlikely to be related to the antecedents (e.g., volitional wrongdoing), moderators (e.g., authoritarianism, efficacy at punishment) and mediators (e.g., negative moral judgments, desire to punish wrongdoers) that we investigate in our work. Retributive philanthropy thus represents a theoretical advance by expanding the range of situations, emotions, and motives that can drive prosocial behavior. In doing so, we answer Labroo and Goldsmith's (2021) call for research that explores the “dirty underbelly” of prosocial behavior.

Substantively, we demonstrate the marketing relevance of retribution in prosocial contexts by exploring features of retributive appeals and retributive donors that could guide charitable marketers in their decision-making. Specifically, we demonstrate that volitional wrongdoing, the efficacy of punishing a wrongdoer, and authoritarianism make retributive donations more appealing to consumers. Collectively, our findings inform charitable marketers that retributive donation options have the potential to increase overall donations and attract new donors.

## Conceptual Framework

### Retributive Philanthropy Is Prosocial Behavior

Contemporary work defines prosocial behavior as actions intended to benefit others or society at some cost to the self (Small and Cryder 2016; White, Habib, and Dahl 2019). Based on this definition, we argue retributive philanthropy is prosocial behavior. All retributive cases in Table 1 involve consumers bearing a personal and financial cost through their donation to a cause that benefits a greater good.

However, retributive philanthropy differs from traditional prosocial behavior in that it involves punishing others. Is such an ulterior motive consistent with prosociality? Indeed, a range of theoretically distinct ulterior motives have been identified as underpinning prosocial behavior (Batson 2022). For example, prior work has found that prosocial behavior is often motivated by a variety of self-interested aims, such as impression management, happiness, life satisfaction, and financial benefits (Curry et al. 2018; Hui et al. 2020; Kristofferson, White, and Peloza 2014; Peloza and Steel 2005). Whether an individual donates for a tax break or to look generous is immaterial to classifying the action as prosocial or not. Therefore, whether an individual donates to humiliate or otherwise harm another has no bearing on the prosocial nature of the donation itself.

Nevertheless, some may argue that retributive philanthropy is morally distinct from self-interested donations by virtue of its harmful nature. On this view, harming others is *immoral*, and such acts should not be prosocial by definition. We contend this argument fails on three grounds.

First, self-interested donations are considered by many to be immoral, insofar as the use of moral engagement for self-promotion corrodes public discourse, fosters political conflict, and is leveraged by individuals with undesirable personality traits like psychopathy and narcissism (Grubbs et al. 2019; Tosi and Warmke 2016). Yet self-interested donations are still treated as prosocial behavior in contemporary literature. Retributive philanthropy should be treated similarly as prosocial behavior that is driven, in part, by nonaltruistic motives.

Second, classifying behaviors in Table 1 as immoral fails to consider the individual beliefs, values, and expectations of donors, which are key to determining prosociality. To illustrate, consider two donors: one who donates to a prochoice charity to protect the reproductive rights of women, and one who donates to a prolife charity to protect the rights of unborn children. Both donors believe they are doing good, despite representing mutually exclusive value systems. By the field's standards, both are engaged in prosocial behavior, because they *intend* to do good, regardless of whether this “good” impact materializes or has negative consequences for others (Small and Cryder 2016; White, Habib, and Dahl 2019). We argue this logic applies similarly to retributive philanthropy: from a retributive donor's perspective, their *motives* are righteous, their *actions* are laudable, and punitive *outcomes* are desirable. Thus, to donors, retributive philanthropy is prosocial.

Finally, retribution is frequently acknowledged as prosocial in adjacent fields of history, philosophy, and psychology. Recent work by feminist and political philosophers has stressed the importance of outrage, protest, and punishment as key factors in remedying injustices (Cherry 2021; Van Doorn, Zeelenberg, and Breugelmans 2014). Retribution can also be prosocial, because it maintains valuable social norms and encourages cooperation (Jackson, Choi, and Gelfand 2019; Sommers 2022). Thus, we contend retributive philanthropy's punitive component may also be prosocial.

### What Makes Retributive Philanthropy Different?

Retribution is, put simply, punishment in response to perceived volitional wrongdoing (Feather 2005; Sommers 2016). Leveraging this definition, we define retributive philanthropy as *charitable acts taken to punish a volitional wrongdoer*. We draw on the retribution literature and abductive analysis from a qualitative study to develop hypotheses about the situations, traits, and features of donation appeals that ought to predict retributive donations. We further make predictions regarding the emotions, thoughts, and judgments that should be associated with retributive donation. We focus on elements of retributive behavior that depart from elements of traditional prosocial behavior explored in prior research.

#### What situations elicit retributive philanthropy?

With retributive philanthropy, we propose that perceived volitional wrongdoing is a key antecedent. What does it mean for an action to be wrong and volitional? Wrongdoing could mean many things, from “forbidden” action (Gold, Pulford, and Colman 2015), an intuitive judgment based on disgust (Wheatley and Haidt 2005), or the violation of a sacred norm or taboo (Tetlock 2002). What unites different types of wrongdoing judgments is a perceived violation of one's moral values (Kähr et al. 2016); strong other-condemning moral emotions such as anger, contempt, and disgust (Lindenmeier, Schleer, and Pricl 2012); and a subsequent desire for punishment. Taken together, we propose moral wrongdoing plays a critical role in eliciting retributive donations.

However, not all wrongs are equal; retribution is not merely concerned with the amelioration of wrongs but also the *volition* of wrongdoers. The extent to which one's behavior is volitional matters a great deal when forming inferences about whether an individual has acted with malicious intent, has harmed others, and is blameworthy for their behavior. In other words, before one can make moral inferences about others, one must first establish that they had some degree of control over their behavior—that the behavior was voluntarily enacted (Williams 1981). Wrongdoing that is volitional is then considered “worse” and more severe than accidental or involuntary wrongs (Ames and Fiske 2013). Individuals witnessing such wrongdoing are then motivated to act as “prosecutors” (Tetlock 2002), seeking to preserve valuable social norms by punishing violators with actions like reprimands (Przepiorka and Berger 2016), physical violence (Fitness 2001), or public shaming (Klonick 2016). Thus, when consumers are exposed to volitional (vs. nonvolitional) wrongdoing, we propose they will be more likely to make retributive donations and do so in larger amounts. Formally,

**H_1a_:** Consumers exposed to volitional (vs. nonvolitional) wrongdoing are more willing to donate to charities that punish the wrongdoer.
**H_1b_:** Consumers exposed to volitional (vs. nonvolitional) wrongdoing donate in greater amounts to charities that punish the wrongdoer.

Volitional wrongdoing should elicit strong negative moral judgments, other-condemning moral emotions, and a desire to punish the wrongdoer (Jackson, Choi, and Gelfand 2019). In other words, when consumers perceive others as deliberately doing wrong, they perceive them as more harmful, having engaged in their behavior with malicious intent to harm others, and blameworthy for their behavior, which in turn elicits a desire to cause some negative state (through punishment) in the wrongdoer. Volitional wrongdoing will also elicit emotions that previous research has associated with the condemnation of others such as contempt, anger, and disgust (CAD; e.g., Haidt 2001). Thus, we expect that the following will characterize retributive (but not traditional) donations:

**H_2a_:** Negative moral judgments increase retributive donations.
**H_2b_:** Stronger (vs. weaker) desire to punish a wrongdoer increases retributive donations.
**H_2c_:** Other-condemning moral emotions associated with a wrongdoer increase retributive donations.

Support for these serial mediation hypotheses would provide evidence that retributive mechanisms are capable of driving prosocial behavior, and that the behaviors we observe are not explained by tribalist or hedonistic accounts. Conceptually, these processes diverge considerably from past prosocial research demonstrating that positive experiences like gratitude (Goenka and Van Osselaer 2019) and love (Cavanaugh, Bettman, and Luce 2015) lead to prosocial actions. Indeed, situations where volitional wrongdoing is perceived and accompanied by other-condemning moral emotions, negative moral judgments, and a desire to punish should invite retributive donations.

#### What type of donors are attracted to retributive philanthropy?

To further support our proposition that philanthropy can be retributive, we predict that donors with vengeful personalities will be more inclined toward punishment of wrongdoers (Tetlock 2002). Contemporary work suggests that positive personality traits like agreeableness (Caprara, Alessandri, and Eisenberg 2012), benevolence (Caprara and Steca 2007), and empathy (Alessandri et al. 2009) predict prosocial behavior. In contrast, we predict that lower agreeableness, higher aggressiveness, and generalized anger, all of which are encompassed within the personality trait of authoritarianism, will positively predict retributive philanthropy. Authoritarianism is traditionally associated with antisocial behavior and inversely associated with prosocial behavior (Costello et al. 2022; Saleem et al. 2017), thus making it an ideal candidate for demonstrating the role of retribution in prosocial behavior. We predict that those higher in authoritarianism will be more predisposed toward punishment of volitional wrongdoers and will, therefore, be more likely to engage in retributive philanthropy:

**H_3_:** Individuals higher (vs. lower) in authoritarianism are more willing to engage in retributive philanthropy when volitional wrongdoing is perceived.

#### What elements of donation appeals elicit retributive philanthropy?

If the goal of retributive philanthropy is to harm a perceived wrongdoer, then the degree to which retributive donations successfully punish them should predict willingness to make retributive donations. Prior work has shown that consumers are more likely to donate when donations are perceived as more effective at helping others (Gneezy, Keenan, and Gneezy 2014) and are more impactful (Cryder, Loewenstein, and Seltman 2013; Sharma and Morwitz 2016). Donors are overhead-averse, preferring charities that put lower proportions of their funds toward fundraising or administrative costs (Gneezy, Keenan, and Gneezy 2014). We extend this work by proposing that donation efficacy, more broadly, refers to how effective a donation is at satisfying a donor's motives. With retributive philanthropy, the primary motive is to harm a wrongdoer. Thus, we predict that consumers who hold negative moral judgments and a desire to punish a wrongdoer will find donation options that are effective at punishing the wrongdoer more appealing. Formally,

**H_4_:** Retributive donations perceived as more (vs. less) effective at punishing a volitional wrongdoer are more appealing to consumers.

If supported, this efficacy-based moderation hypothesis would further bolster our proposition that philanthropy can be retributive. Theoretically, this finding would both parallel and depart from existing findings in the prosocial behavior literature, as no research to date has shown that efficacy at punishing others can positively influence donations.

## Overview of Studies

**Figure 1.**
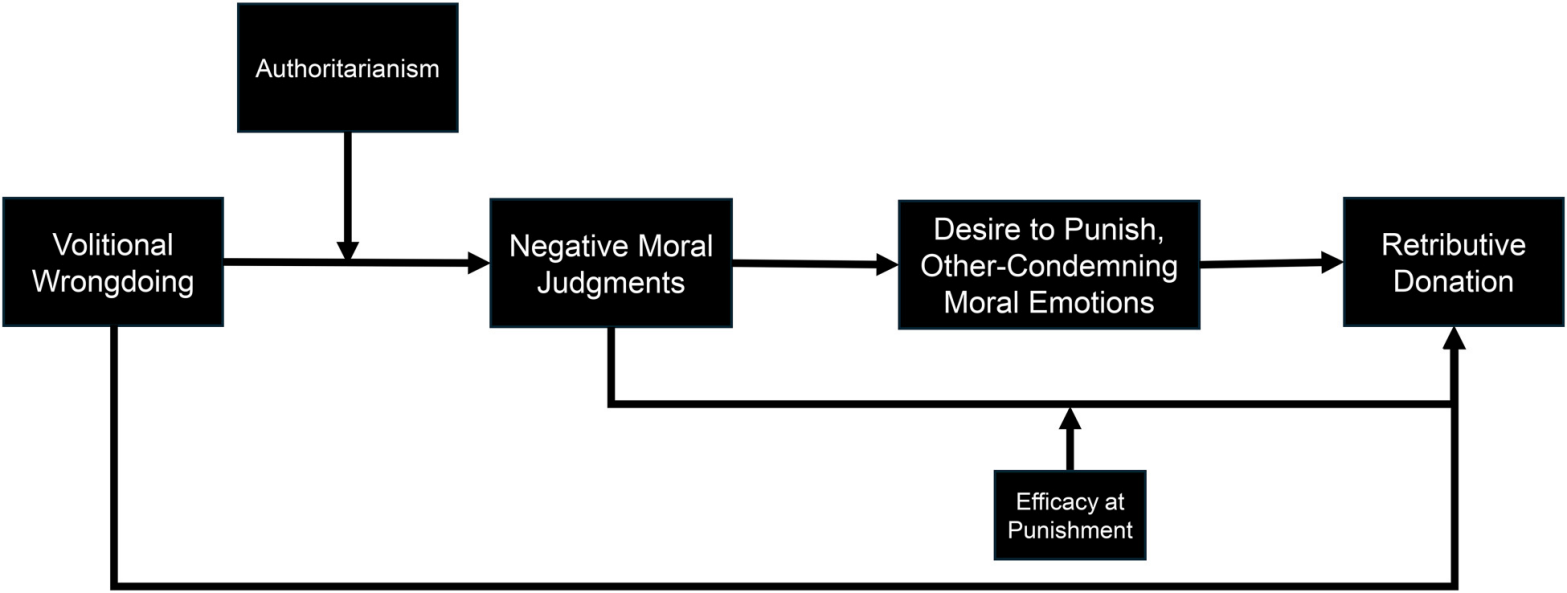
Conceptual Framework.

We present 12 studies (7 primary, 5 supplementary; see Web Appendix A) that leverage a mixed-methods approach and multiple real-world examples to examine the phenomenon of retributive philanthropy, the opportunity it provides to charitable marketers, and the role that retribution can play in increasing philanthropic behavior. Figure 1 provides a visual overview of our proposed conceptual framework, and Table 2 provides a summary of our primary studies.

**Table 2.** Overview of Studies.

**Study 2: Real-World Evidence**	**Design:** 2 (GoFundMe mentioned vs. GoFundMe not mentioned) **DV:** Amount donated **Focal test:** T-test between conditions **Focal result:** Between-group difference = $23.43, t(4,064.49) = 5.22, *p *< .001			
	**GoFundMe Mentioned**		**GoFundMe Not Mentioned**	
Donation amount	$110.89		$87.46	
**Study 3: Volitional Wrongdoing and Retributive Benefits**	**Design:** 2 (wrongdoing: volitional vs. nonvolitional) × 2 (retributive option: present vs. absent) **DV:** Choice to donate cash to retributive charity, donate to nonretributive charity, or keep cash **Focal test:** Multinomial logistic regression on choice between charities and not donating **Focal result:** Volition × Retributive option = .93, SE = .28, 95% CI: [.18, 1.69], *p *= .015			
	**Volitional**		**Nonvolitional**	
	**Option Present**	**Option Absent**	**Option Present**	**Option Absent**
Retributive charity choice	41.52% (retributive option)	37.07%	33.33% (retributive option)	51.30%
**Study 4: Psychological Mechanisms**	**Design:** 2 (wrongdoing: volitional vs. nonvolitional) × 2 (identity: Jewish vs. non-Jewish) **DV:** Likelihood of retributive donation **Focal test:** Mediation with negative moral judgments and desire to punish (preregistered) **Focal result:** Index of serial mediation = .02, SE = .006, 95% CI: [.009, .033]			
	**Volitional**		**Nonvolitional**	
	**Jewish**	**Non-Jewish**	**Jewish**	**Non-Jewish**
Donation likelihood	3.65	3.10	3.63	2.78
**Study 5: Other-Condemning Moral Emotions**	**Design:** 2 (wrongdoing: volitional vs. nonvolitional) **DV:** Likelihood of retributive donation **Focal test:** Mediation with negative moral judgments and other-condemning moral emotions (preregistered) **Focal result:** Index of serial mediation = .255, SE = .039, 95% CI: [.186, .336]			
	**Volitional**		**Nonvolitional**	
Donation likelihood	2.67		1.83	
**Study 6: Donor Personalities**	**Design:** 2 (sample: left-wing vs. right-wing) × 2 (norm violation: left-wing vs. right-wing) **DV:** Likelihood of retributive donation **Focal test:** Moderation by RWA and LWA (preregistered) **Focal results:** LWA × Left violation = .610, t(396) = 3.58, *p *< .001; RWA × Right violation = .997, t(390) = 6.33, *p *< .001			
	**Left-Wing Sample**		**Right-Wing Sample**	
	**Left-Wing Norm Violation**	**Right-Wing Norm Violation**	**Left-Wing Norm Violation**	**Right-Wing Norm Violation**
Donation likelihood	3.49	2.07	1.90	3.85
**Study 7: Efficacy at Punishment**	**Design:** 2 (wrongdoing: volitional vs. nonvolitional) × 2 (punishment efficacy: high vs. low) **DV:** Likelihood of retributive donation **Focal test:** Mediation by negative moral judgments, moderation by punishment efficacy (preregistered) **Focal result:** index of moderated mediation = .329, SE = .096, 95% CI: .145, .519			
	**Volitional**		**Nonvolitional**	
	**High Punishment Efficacy**	**Low Punishment Efficacy**	**High Punishment Efficacy**	**Low Punishment Efficacy**
Donation likelihood	2.79	2.44	1.94	1.90

## Study 1: Qualitative Interviews

In Study 1, we took an exploratory qualitative approach to deeply investigate the motives, emotions, and experiences of retributive donors and their targets. Because no prior work on this phenomenon exists, this approach was critical to enable a rich, open-ended exploration (Schnurr et al. 2022; Whitley et al. 2022) and to bridge the philosophical, psychological, and marketing literatures that we theorize relate to retributive philanthropy. We conducted ten interviews with self-identified retributive donors and targets with two aims: (1) to identify if retributive philanthropy empirically aligns with key elements of retributive and prosocial behaviors, and (2) to compare and contrast participants’ experiences of retributive philanthropy with traditional prosocial behaviors. To achieve these aims, we asked participants to describe their target or retributive donor, the retributive donation, and their feelings throughout the experience.

### Participants

We interviewed participants who made or were targeted by retributive donations. Six participants were recruited via TikTok, a platform used by researchers to investigate hard-to-reach, niche, or otherwise marginalized communities (MacKinnon, Kia, and Lacombe-Duncan 2021). Four participants were recruited via email (see Web Appendix B for recruitment message). Our sample represents a diversity of genders, education levels, ages, careers, political perspectives, and retributive philanthropy cause areas (see Table 3). We sought diverse perspectives to identify elements of the phenomenon that either depend on or transcend participant demographics.

**Table 3.** Study 1 Participant Demographics.

**Nickname**	**Gender**	**Age Group (Years)**	**Education**	**Job**	**Region**	**Role**
Emma	Female	25–34	Master’s	Student	Canada	Donor
Alice	Female	35–44	Undergraduate	Service	UK	Donor
Abigail	Female	35–44	Master’s	Design	USA	Donor
Cynthia	Female	18–24	Undergraduate	Temp	USA	Donor
Eunice	Female	25–34	Master’s	Marketing	UK	Donor
Irma	Nonbinary	18–24	Undergraduate	Student	UK	Donor
Steven	Male	25–34	High school	Streamer	USA	Target and donor
Ian	Male	25–34	Undergraduate	Streamer	USA	Target and donor
James	Male	25–34	Master’s	Photography	Canada	Donor
Max	Male	25–34	Undergraduate	Artist	USA	Target

### Interviews

Each interview lasted 20 to 30 minutes on average (McCracken 1988). Because participants were contacted online and from diverse regions, we interviewed them via Zoom or Discord. The researcher's camera was always on, and all participants elected to be visible. With participants’ consent, interviews were audio-recorded and immediately transcribed postinterview (see Web Appendix B for interview guide).

There was significant variance in how participants pursued or experienced retributive philanthropy. Some made donations in others’ names to charities their targets would dislike. Others made or were targeted by donations to charities that were in opposition to the target's values. Some used the platform afforded by a donation to communicate hurtful messages. Despite this variance, the key themes we observed remained consistent across participant experiences.

### Analysis

We analyzed and coded interviews to identify key themes in participants’ experiences of retributive philanthropy and to understand how it compared to more traditional prosocial behaviors. This was done in an iterative process, incorporating theoretical insights from each interview to insights gleaned from prior interviews until theoretical saturation was reached (Miles, Huberman, and Saldaña 2014; Spiggle 1994). We identified several key themes that transcended participants’ individual characteristics and were present in all cases of retributive philanthropy. This analysis was performed abductively with reference to the retribution literature to identify commonalities with previously explored themes of retribution. Participants’ experiences with retributive philanthropy were universally characterized by (1) perceptions of volitional wrongdoing committed by a target, (2) strong other-condemning moral emotions, and (3) a desire to punish. Further, retributive donors universally reported that retributive philanthropy was sharply distinct from traditional prosocial behaviors. We outline each theme in turn.

#### Volitional wrongdoing and negative moral judgments

For retributive donors, their targets were universally characterized as having voluntarily committed a wrongful act, leading to negative judgments of targets such as “racist” or “[an] a**hole.” Importantly, these were not minor harms; no donor reported mere annoyance or irritation. Instead, they reported experiencing or witnessing harms extreme enough to warrant an aggressive response (Marwick 2021). For example, Cynthia described her experience of being sent to a conversion therapy camp by her aunt:

I was with a lot of my peers being held in a room and getting yelled at for hours on end, saying that we need to repent our sins and that being gay is a sin. (Cynthia)

Cynthia ultimately made donations to LGBTQ+ charities in the name of homophobic family members and included their real addresses to ensure that they received notice of the donation, with the understanding that her donations would upset them.

#### Other-condemning moral emotions

Participants reported feeling some type of anger (e.g., “anger,” “fury,” “pissed off”) as a result of experiencing or witnessing a wrongdoing. This aligns with the literature on negative moral judgments leading to anger (Antonetti 2016; Malle 2021). Participants also reported ruminating on their emotions and negative moral judgments of their target, which is consistent with other forms of retributive behavior (Kähr et al. 2016).

#### Desire to punish

Participants then pursued punishment of the wrongdoer in a manner they described as vengeful, retributive, or punitive, often using language like “karma,” “offset,” or “revenge.” Accordingly, the donations offset the target's wrongdoing in a poetically just way, matching the retribution to the offense. Their reported experiences generally tracked the “eye for an eye” mindset that characterizes retributive behavior:

I’m a big equal exchange kind of guy. … And I love fighting with people. It's a lot of fun for me. … So if someone wants to attack me, I love it because I get to attack them back, but I try not to attack people if they don’t attack me first. (Steven)

Importantly, many participants said that the potential for retribution was an essential element in choosing to donate (e.g., Cynthia stated, “I would not have donated if I could not put [family members’] names on it”). In cases where retribution was not the sole motivation for donating, participants reported that it made them donate sooner. These themes support our proposition that retributive philanthropy can potentially elicit new donations over and above traditional processes described in the prosocial behavior literature (e.g., White, Habib, and Dahl 2019).

We found convergent evidence for this notion in our interview with James, who created a book of Ukrainian vistas titled Russian Warship, Go F**k Yourself (2022). James described how making all proceeds go toward Ukrainian charities made the retributive message feel more satisfying for consumers, but that the “go f**k yourself” message was a primary sales driver:

I don’t think we would have nearly a fraction of the sales if we didn’t have both of those things like the “Russian Warship Go F**k Yourself” … messaging gives it traction, and the feel-good support Ukraine idea is … what makes people hit “buy.” (James)

In summary, there is evidence in this qualitative data that donations can be retributive, consistent with the characteristics of retribution observed in the literature. Targets were perceived as volitional wrongdoers and subsequently judged as blameworthy, which is a critical component of negative moral judgments (Jackson, Choi, and Gelfand 2019; Malle 2021). In addition to experiencing other-condemning negative emotional states (e.g., of the CAD trio of contempt, anger, disgust), participants expressed a desire to punish their targets, which motivated their donations. Importantly, participants’ descriptions of these states were distinct from emotions traditionally associated with prosocial behavior like moral elevation or love (Algoe and Haidt 2009; Cavanaugh, Bettman, and Luce 2015).

#### Comparison with traditional donations

As expected, participants’ experiences with traditional donations matched well with extant literature on prosocial behavior. Most reported making donations that helped others. By contrast, their retributive donations were described as more concerned with achieving justice and involved other-condemning moral emotions like anger. Taken together, we find that the overall experience of retributive philanthropy is clearly distinct from traditional prosocial behaviors in terms of emotions, motives, and behaviors.

### Discussion

In these interviews, we observed points of similarity and points of departure from retribution and prosocial literatures. Retributive donations were similar to prior accounts of retribution and distinct from prior accounts of prosocial behavior in that they involved negative moral judgments (Jackson, Choi, and Gelfand 2019; Sommers 2016), other-condemning moral emotions, a desire to punish, and actively ensuring that punishment was realized. These observations mirror findings that donors are motivated by donation efficacy (White, Habib, and Dahl 2019). While the phenomenon of retributive philanthropy shares conceptual similarities with retribution and prosocial behavior, these results suggest it is also conceptually distinct and that it can occur in the context of large-scale campaigns and individual giving. Study 2 finds convergent themes of anger at volitional wrongdoing in a large-scale retributive campaign with real donations.

## Study 2: Real-World Evidence

Study 2 provides real-world evidence for retributive philanthropy in a large-scale donation context. Consistent with the findings of Study 1, we show that perceived volitional wrongdoing and negative moral judgments are critical to retributive donations. Our context was a campaign for a viral protest movement. In January 2022, a group of Canadian truckers occupied cities and blockaded highways to pressure the government to rescind COVID-19 policies. GoFundMe, a crowd-funding platform, hosted “Freedom Convoy 2022,” a campaign that raised millions of dollars to help protesting truckers refuel, eat, and supplement their lost income. In response, the Canadian government froze protestors’ bank accounts, and GoFundMe subsequently removed the campaign from the platform and refunded donations (McDade 2022). Organizers and donors quickly moved to another donation platform called GiveSendGo and raised $9 million in under 24 hours. Supporters expressed outrage at the campaign removal from GoFundMe, indicating that they *perceived wrongdoing* by GoFundMe and the Canadian government.

The GoFundMe campaign removal and subsequent shift to GiveSendGo presented the opportunity to investigate how donors react to volitional wrongdoing. Specifically, both platforms allowed donors to provide comments to accompany their donations, and an examination of the comments suggests that donors perceived the GoFundMe campaign freeze to be morally wrong—describing it in morally loaded terms like “disgusting,” “corruption,” and “betrayal”—and deliberately enacted (e.g., the government “colluded with GoFundMe to defraud donors”). These perceptions of volitional wrongdoing were experienced and communicated above and beyond a desire to support the truckers or seek revenge against the government. Regardless of donors’ retributive desires during the initial GoFundMe campaign, there was clearly *greater* cause for retribution after it was disbanded. We leverage this (perceived) wrongdoing to see whether donors increase donation amounts when wrongdoing is volitional and more salient.

### Method and Results

We tested our hypothesized effect by investigating the donations of 100,270 individuals who gave to the GiveSendGo campaign. This dataset included information about donors’ chosen pseudonyms, their donation amounts, and any comments associated with their donation. We expected that moral outrage would be highest among donors for whom the GoFundMe campaign freeze was salient and that such donors would make larger donations.

We algorithmically classified each donation according to whether its associated comment contained the strings “go,” “fund” or “fraud,” and “me” (case-neutral) to accommodate different ways of spelling or referring to “GoFundMe” (e.g., “gofund me,” “GoFraudMe”). Of the 108,012 GiveSendGo donations, 2,037 (1.89%) mentioned GoFundMe. To validate that the GoFundMe campaign removal was perceived as a volitional wrongdoing, donor comments were classified as containing moral outrage or not using a moral outrage text classification algorithm (Brady et al. 2021). Moral outrage is a response evoked when volitional wrongdoing is perceived (Jackson, Choi, and Gelfand 2019) and, thus, provides a good proxy for perceived volitional wrongdoing in our dataset. Overall, 6,836 (6.33%) of comments were identified as containing moral outrage. Example comments referencing GoFundMe and exhibiting outrage are provided in Table 4; importantly, these comments often included calls to shame, denounce, or otherwise punish the government, which was perceived as morally responsible for violations of donors’ freedoms.

**Table 4.** Examples of Comments Referencing GoFundMe That Contained Moral Outrage.

	**Comment**
$25	This donation is of dual intent. 2 support to good cause of Truckers Freedom Convoy and 2 denounce Go Fund Me as puppets who cannot be trusted to handle trust & money. 2 those at Go Fund Me I extent my right middle finger 2 U in disgust, contempt & derision 4 this fraud, bait & switch scame & their betrayal of those trusting them with their money. I foist upon them all the ill will I can extent.
$50	Oh Canada, So Glorious and (ToBe) FREE! thank u truckers as defined by courage & morality, & all who u have inspired to seek freedom.I am doubling my GoFundMe pledgge because my blood is boiling: how dare they steal from Freedom Lovers. I am American, but Canadian blood runs through my veins: Mom's fr Ottawa bacl to 1600's and Dad is from Quebec: I am a first generation American. Viva la Canada!
$150	My $75 to GoFundMe was rejected, so I’ve doubled-down with $150. If we all double-up, we’ll hit 20 million. The forced vax left me with permanent (so far 8 months) neurological damage. The jab don’t work- ‘vaxxed’ still get/spread COVID, end up in the hospitals on ventilators or in the funeral parlour. Trudeau wants to force pain and suffering upon people and blame ‘unvaxxed’ truckers. Freedome!

In line with our predictions, comments mentioning GoFundMe were four to five times more likely to contain moral outrage relative to those that did not (26.07% vs. 5.95%; Δ = 20.12%, CI_95_: [18.18%, 22.05%], χ^2^ = 1,361.1, *p *< .001). These results suggest that donors perceived the government's actions to be a volitional wrong, thus meriting retribution. Importantly, we empirically observed that larger donations were made by donors mentioning GoFundMe (M_donation_ = $110.89) compared with donors who did not (M_donation_ = $87.46; Δ = $23.43, CI_95_: [14.63, 32.23], t(4,064.49) = 5.22, *p *< .001). Taken together, these results support H_1b_ and H_2c_, suggesting that exposure to volitional wrongdoing motivates larger retributive donations and that anger, an other-condemning moral emotion, is characteristic of retributive donations.

### Discussion

The results of Study 2 suggest that situations conducive to retribution can result in larger donations (H_1b_). Specifically, donors who perceived the GoFundMe freeze to be morally wrong and mentioned it made larger donations. Using real-world data, we demonstrate support for retributive donations in general, as well as our hypotheses (H_1b_, H_2c_). In addition, observing these results in this context shows that retributive philanthropy is not limited to liberal causes, as the Freedom Convoy movement was primarily supported by conservatives (McDade 2022).

This study also revealed themes of retributive donation consistent with Study 1. We observed that for donors for whom a wrongdoing was salient (mentioned GoFundMe), there were more expressions of outrage and anger and some associated differences in donation amounts. In subsequent studies, we provide a controlled causal account of the role of volitional wrongdoing, as well as consequent negative moral judgments and desire to punish, on retributive donations.

## Study 3: Volitional Wrongdoing and Retributive Benefits

In Study 3, we examine the causal effect of volitional wrongdoing on retributive donation (H_1a_), as well as the effect of an organization's choice to offer a retributive benefit in their donation appeal. Specifically, we expect that retributive donation options are most appealing to consumers when volitional (vs. nonvolitional) wrongdoing has occurred.

### Method

Five hundred forty-eight undergraduate participants (M_age_ = 18.96 years; 43.25% female, 55.66% male, 1.09% nonbinary/genderqueer/gender neutral) from a large North American university completed this study in exchange for course credit and were randomly assigned to conditions in a 2 (wrongdoing: volitional vs. nonvolitional) × 2 (retributive option: present vs. absent) between-participants design (see Web Appendix C for measures and stimuli across studies).

Participants first read an article where a professor either (1) deliberately used a racial slur (the N-word) or (2) said a word that sounds like a racial slur (the Chinese word “nèige”), adapted from a true story reported by Flaherty (2020), but with locations and names changed (see Web Appendix D for a posttest of this manipulation). To maintain the cover story, participants shared their opinion about the situation in an open-ended response.

Participants were then given a $1 cash bonus. They had the option to keep the $1 or to donate to one of two fictitious charities with relevant cause areas (adapted from Goenka and Van Osselaer [2019]). Both charities (Kentucky Antiracist Student Alliance [KASA] and Western Kentucky Black Students [WKBS]) focused on promoting antiracism. In the retributive-option-present condition, one of the organizations (KASA) also promised to send a letter to the dean calling for the professor's dismissal for every donation received—a consequential punishment. In the retributive-option-absent condition, both organizations promised to promote antiracism, but no act of retribution was mentioned.

Upon learning of the options, participants chose between keeping their bonus or donating to one of the two organizations. We predicted an interaction between our experimental conditions, such that participants would donate at greater rates to KASA when (1) the organization offered a retributive benefit and (2) the professor's wrongdoing was volitional.

### Results

We analyzed our data using a multinomial logistic regression to predict donation choice using the control charity (WKBS) as the base value. This analysis allowed us to directly test the relative appeal of our retributive charity (KASA) to more traditional options. The two sets of coefficients in Table 5 represent participants’ likelihood of choosing either donations to KASA or keeping their bonuses instead of donating to WKBS.

**Table 5.** Multinomial Logistic Regression of Donor Choice.

**Parameter**	**β**	**SE**	**95% CI**	**z**	***p***
**Donate to KASA**					
(Intercept)	.05	.19	[−.31, .42]	.28	.80
Volition	−.58	.27	[−1.11, −.06]	−2.17	.030
Retributive option	−.75	.28	[−1.29, −.20]	−2.70	.007
Volition × retributive option	.93	.39	[.18, 1.69]	2.42	.015
**Keep the Bonus**					
(Intercept)	−.81	.24	[−1.28, −.34]	−3.35	<.001
Volition	−.59	.36	[−1.29, .11]	−1.66	.10
Retributive option	−.25	.33	[−.91, .40]	−.75	.50
Volition × retributive option	.55	.49	[−.41, 1.51]	1.13	.30

First, offering a retributive option negatively affected donations to KASA when the professor's wrongdoing was not volitional (β = −.75, SE = .28, CI_95_: [−1.29, −.20], *p *= .007). This result is unsurprising, as consumers generally do not want to punish others who have done nothing wrong. Central to our theorizing, we found a significant interaction between volitional wrongdoing and the presence of a retributive option (β = .93, SE = .39, CI_95_: [.18, 1.69], *p *= .015), such that when KASA offered a retributive benefit and the professor's wrongdoing was volitional, participants were more likely to donate to KASA.

### Discussion

The results of Study 3 support our conceptualization of retributive philanthropy and highlight the importance of perceived volitional wrongdoing as a precursor to retribution (H_1a_). We show that enabling retribution can be leveraged to drive donations, a behavior distinct from more traditional forms of retribution such as admonishments or violence.

These results also offer valuable managerial insights. First, we demonstrate that charities can benefit from offering retributive donation options to consumers. In circumstances where volitional wrongdoing is perceived, retributive donation options attract new donors relative to traditional options. Second, we qualify this finding and advise charities to ensure that donors actually perceive a wrongdoing as volitional before leveraging retributive donation appeals.

Having established a causal effect of volitional wrongdoing on retributive donation choice, we next test our proposed process that negative moral judgments (proximal mediator) and desire to punish (distal mediator) explain the effect of volitional wrongdoing on retributive donation. Observing this process would further support a retributive account of our phenomenon, as retribution is concerned with the punishment of volitional wrongdoers.

## Study 4: Psychological Mechanisms for Retributive Philanthropy

The goal of Study 4 was to provide process evidence for the effect of volitional wrongdoing on retributive donation. We propose that perceiving volitional wrongdoing influences retributive donations, because it is seen as morally worse and thus heightens one's desire to punish. We test this serial mediation account in Study 4 (H_1a_, H_2b_) using another current and relevant context—specifically, controversy over Kanye West's antisemitic statements. This controversy is well-suited to analysis of volitional wrongdoing and retributive behavior, as there is debate over whether West's antisemitism is attributable to his bipolar disorder (Levitz 2022). To examine our predictions, we manipulated whether participants were informed that West's antisemitism was or was not caused by his mental health problems. Harmful actions attributable to mental illness are seen as less volitional (i.e., not under one's control), less reflective of one's character, and less deserving of punishment (Finkel and Slobogin 1995; Mercier, Norris, and Shariff 2018). We, therefore, expect that when West's statements are seen as unrelated to his mental health (i.e., more volitional), participants will be more punitive toward him and subsequently more likely to make a retributive donation.

We also test a potential alternative explanation for retributive donations: identity threat. An identity-threat account might suggest that consumers who are most affected by a volitional wrongdoing would be most likely to make a retributive donation (e.g., White and Argo 2009). Our retribution account predicts that consumers will make retributive donations when they perceive wrongdoing as volitional, and that this increase in donation will result even among consumers who are not personally affected by wrongdoing. We test this alternate account by recruiting Jewish and non-Jewish participants. An identity threat account of our phenomenon would predict a significant interaction between volitional wrongdoing and identity, such that when identity is relevant (i.e., Jewish population), retributive donations would be higher when a wrongdoing is volitional, but that no differences would emerge when identity is not relevant. However, our retribution account would predict only a main effect of volitional wrongdoing such that retributive donations would be higher when volitional wrongdoing was present versus absent.

### Method

Prolific Academic participants (N = 1,198; ages 18–79 years, M_age_ = 33.42 years; 51.17% female, 48.33% male, .50% prefer not to answer) completed this study in exchange for financial payment and were randomly assigned to conditions in a 2 (wrongdoing: volitional vs. nonvolitional) × 2 (identity: Jewish vs. non-Jewish) between-participants design. This study was preregistered at https://aspredicted.org/8znz-nnsp.pdf.

Per our preregistration, our goal was to recruit a balanced sample of 600 Jewish people and 600 non-Jewish people. However, Jewish people are a small minority of Prolific Academic users, and thus we anticipated a risk of falling short of recruiting 600 Jewish participants. We therefore recruited participants in two waves. In our first wave, we recruited only Jewish participants for one week, after which point we halted collection and then recruited non-Jewish participants. We recruited 279 participants in Wave 1 and 919 participants in Wave 2. Of the 919 participants recruited in the non-Jewish wave, 65 (7.07%) identified as either ethnically or religiously Jewish (which they had not self-disclosed on the Prolific platform) and were thus added to the Wave 1 data. Given this low proportion, we note that all results are robust to these Jewish participants being treated as non-Jewish or Jewish in our analyses.

Participants read an article discussing Kanye West's recent antisemitic remarks, which said that experts claim either that (1) West's antisemitism is not attributable to his bipolar disorder (volitional wrongdoing), or (2) West's antisemitism is attributable to his bipolar disorder (nonvolitional wrongdoing) (see Web Appendix C for stimuli; see Web Appendix E for a posttest of this manipulation). We then measured negative moral judgments of West (harm: “I believe Kanye West was antisemitic”; malicious intent: “I believe Kanye intended to be antisemitic”; and blameworthiness: “I blame Kanye for being antisemitic”; three items; 1 = “Strongly disagree,” and 7 = “Strongly agree”; α = .89), and their desire to punish him (adapted from Grégoire, Laufer, and Tripp [2010]; e.g., “I would like to punish Kanye West”; four items; 1 = “Strongly disagree,” and 7 = “Strongly agree”; α = .85).

Finally, participants were presented with a retributive donation option from a charity addressing injustice against Jewish people. Specifically, participants were informed that the Anti-Defamation League (ADL) was soliciting funds to lobby for West's business partnerships to be canceled to financially punish him for his behavior. Participants rated how likely they were to donate to the ADL on a seven-point scale (1 = “Extremely unlikely,” and 7 = “Extremely likely”).

### Results

**Figure 2.**
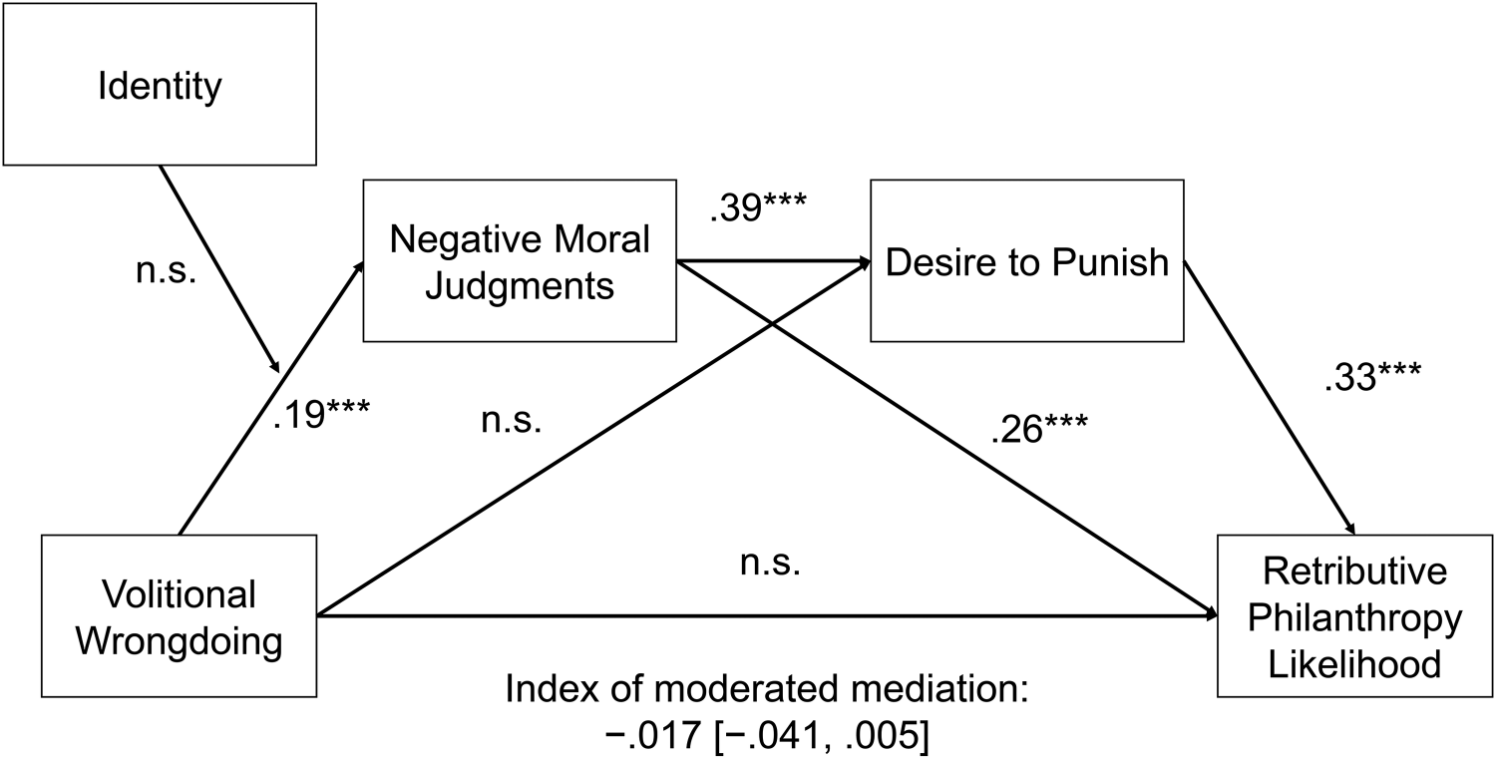
Study 4 Results.

Per our preregistration, we used Hayes’s (2013) PROCESS Model 83 with bootstrap resampling (N = 10,000) to test the mediating pathway from volitional wrongdoing to negative moral judgments, desire to punish, and retributive donation likelihood, with Jewish identity moderating the relationship between our manipulation and negative moral judgments (Figure 2).

We first observed a significant effect of our manipulation on donation likelihood, such that participants in the volitional wrongdoing condition (M = 3.26) expressed greater likelihood of making a retributive donation relative to those in the nonvolitional condition (M = 3.02; t(1,195.7) = 2.28, *p *= .022).

Consistent with our proposed retribution account, we found a significant effect of our experimental manipulation on negative moral judgments (β = .191, SE = .049, CI_95_: [.096, .287], t(1,194) = 3.92, *p *< .001), which in turn affected participants’ desires to punish West (β = .388, SE = .024, CI_95_: [.340, .435], t(1,195) = 15.92, *p *< .001) and, subsequently, increased participants’ likelihood of making retributive donations (β = .331, SE = .040, CI_95_: [.254, .409], t(1,194) = 8.38, *p *< .001). We note that there was no significant direct effect of volitional wrongdoing on desire to punish. We also did not observe a significant interaction between participants identifying as Jewish and our manipulation. Jewish participants were not more sensitive to the volitional nature of Kanye's actions than non-Jewish participants (β = −.136, SE = .091, CI_95_: [−.315, −.050], t(1,194) = −1.48, *p *= .138). The index of moderated mediation in our model did not exclude zero (β = −.017, SE = .012, CI_95_: [−.041, .005]), which suggests that participants’ holding a threatened identity does not elevate the focal retributive effect we observed in this study. A simpler Hayes PROCESS Model 6 model of the effect of volitional wrongdoing mediated by negative moral judgments and desire to punish (unmoderated by Jewish identity) did, however, show a significant index of serial mediation (β = .02, SE = .006, CI_95_: [.009, .033]).

### Discussion

The results of Study 4 provide direct process evidence for the key role that negative moral judgments and desire to punish play in retributive donations in yet another current and relevant context (e.g., antisemitism). Wrongdoing understood as volitional (vs. nonvolitional) is taken as more (vs. less) reflective of an individual's moral character, increasing negative moral judgments and a subsequent desire to punish, which, in turn, increases the likelihood of making a retributive donation to a charity (H_1a_, H_2a–b_). In this study, we also rule out identity threat as an alternative explanation of our observed pattern of results. Finally, we directly replicate our observed serial mediation process in a follow-up preregistered study that did not specifically recruit Jewish participants (Web Appendix F).

## Study 5: Other-Condemning Moral Emotions and Retributive Donations

Thus far, we have accumulated qualitative and quantitative support for the operation of retributive processes and motives in prosocial behavior. In Study 5, we provide additional process support for our retribution account by examining the role of other-condemning moral emotions. Specifically, we sought to identify how other-condemning moral emotions influence the success of retributive donation appeals, while also demonstrating their relation to volitional wrongdoing and negative moral judgments. Prior research has identified the motivating role of positive emotions—such as love (Cavanaugh, Bettman, and Luce 2015)—on donation. In contrast, we predicted that other-condemning moral emotions such as contempt, anger, and disgust will be salient and critical in transmitting the effect of negative moral judgments on retributive donation.

### Method

Prolific Academic participants (N = 1,200; ages 18–80 years, M_age_ = 41.65 years; 50.00% female, 50.00% male) completed this study in exchange for financial payment and were randomly assigned to conditions in a two-factor (wrongdoing: volitional vs. nonvolitional) between-participants design. One participant failed to complete the study in its entirety and was therefore excluded. Our data collection and analysis plan were preregistered at https://aspredicted.org/t5kr-j9q7.pdf. We used the stimuli, manipulations, and retributive donation dependent variable from Study 3. To assess other-condemning moral emotions, participants rated the extent to which the story made them feel anger, contempt, or disgust using a nine-item, seven-point scale (1 = “Strongly disagree,” and 7 = “Strongly agree”; α = .971; adapted from Karppinen, King, and Russell [2023]).

#### Results

We first observed a main effect of our manipulation, such that participants were more likely to make a retributive donation when the professor's wrongdoing was voluntary (M = 2.67) versus accidental (M = 1.83; t(1,109.2) = 8.40, *p *< .001). Consistent with the results of Study 4, we also observed a significant indirect effect of volitional wrongdoing on retributive donation through negative moral judgments (PROCESS Model 4; β = 1.03, SE = .074, CI_95_: [.887, 1.179]).

**Figure 3.**
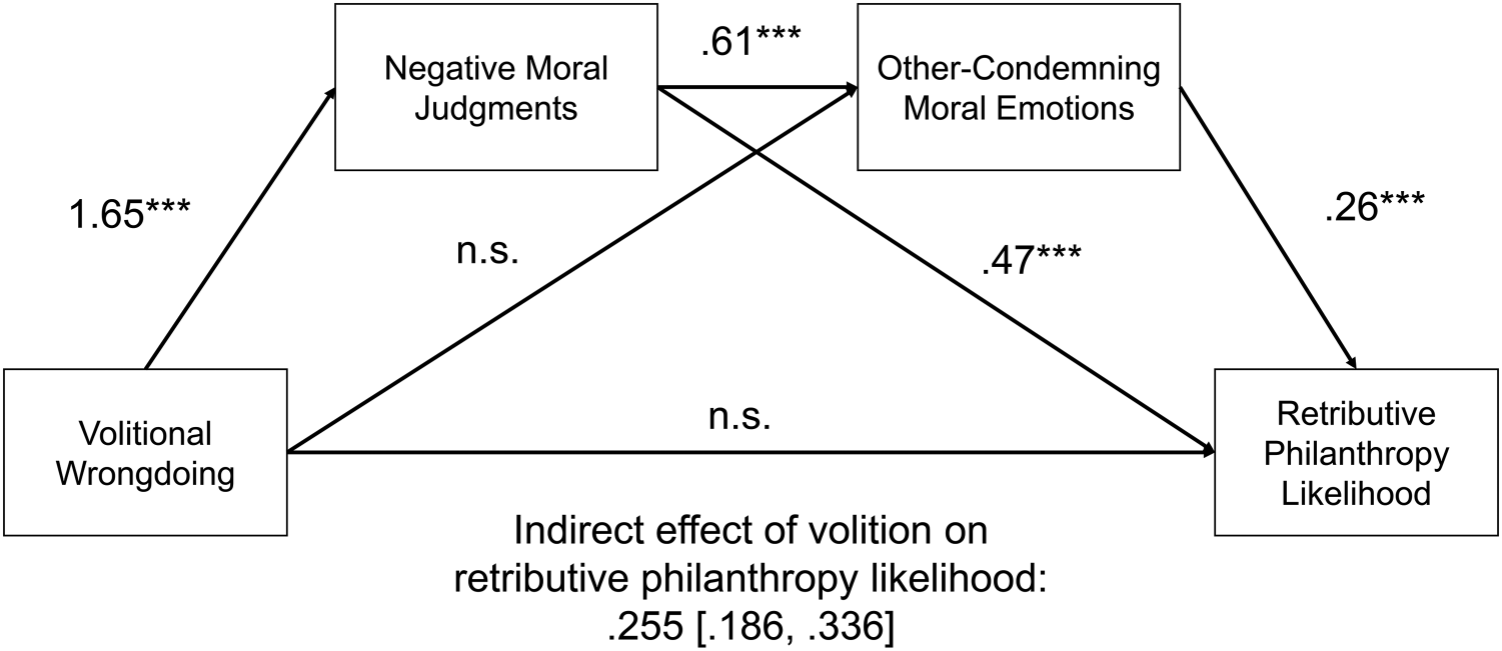
Study 5 Results.

We next tested whether participants’ other-condemning moral emotions mediated the effect of negative moral judgments on retributive donation (PROCESS Model 6, 10,000 resamples). We found that negative moral judgments positively predicted increased other-condemning moral emotions (β = .605, SE = .025, CI_95_: [.557, .654], t(1,196) = 24.60, *p *< .001), which in turn predicted higher likelihood of making a retributive donation (β = .256, SE = .029, CI_95_: [.200, .312], t(1,195) = 8.94, *p *< .001). We did not observe a significant direct effect of volitional wrongdoing on other-condemning moral emotions. The index of serial mediation excluded zero (β = .255, SE = .039, CI_95_: [.186, .336]). Other-condemning moral emotions significantly mediated the relationship between negative moral judgments and donation (see Figure 3).

In several complementary preregistered PROCESS Model 6 analyses, we estimate the mediating effect of each subcomponent of our emotion scale (contempt, anger, disgust) and find consistent results across each, such that contempt (β = .194, SE = .033, CI_95_: [.133, .261]), anger (β = .212, SE = .033, CI_95_: [.152, .279]), and disgust (β = .260, SE = .040, CI_95_: [.186, .345]) all significantly mediated the effect of volitional wrongdoing on retributive donation.

### Discussion

The results of Study 5 support our theorizing that retributive donation, a manifestation of retribution, is related to and explained by other-condemning moral emotions. These results also align with our real-world investigations of retributive philanthropy in Studies 1 and 2, where we observed consistent themes of anger and disgust at perceived wrongdoers.

In the analyses presented, we assume that other-condemning moral emotions follow moral judgments. However, there are other plausible accounts of moral reasoning and emotions that propose that moral emotions precede moral judgments (e.g., Haidt 2001). We therefore test alternate specifications of our proposed model where the order of our mediators is reversed and find consistent results (see Web Appendix G).

## Study 6: Donor Personalities and Willingness to Make Retributive Donations

In Study 6, we provide additional process support for our retribution account via individual-difference moderation. Specifically, we sought to identify whether certain personality traits known to be more retributive-oriented would be more receptive to donating when retribution was paired with donation. Authoritarianism is a personality trait that is traditionally associated with punishment (Altemeyer 2007) and, therefore, likely to be more amenable to retributive donation.

We operationalize authoritarianism using Costello et al.’s (2022) Left-Wing Authoritarianism (LWA) scale and Altemeyer's (2007) Right-Wing Authoritarianism (RWA) scale. We expect left-wing partisans higher in LWA will be more likely to make retributive donations when presented with a violation of left-wing norms and that right-wing partisans higher in RWA will be more likely to make retributive donations when presented with a violation of right-wing norms. Such results would thus constitute evidence for a general effect of authoritarianism on retributive donation, rather than an effect of merely RWA or LWA.

### Method

Prolific Academic participants (N = 794; ages 18–94 years, M_age_ = 45.37 years; 50.13% female, 49.87% male) completed this study in exchange for financial payment and were randomly assigned to conditions in a two-factor (norm violation: left-wing vs. right-wing) between-participants design. Our data collection and analysis plans were preregistered at https://aspredicted.org/xgb4-sn7c.pdf. We recruited two independent samples of left-wing and right-wing partisans in two waves on Prolific Academic. Our first wave of recruitment consisted of 400 self-identified Democrats, while our second wave consisted of 400 self-identified Republicans. Six participants in the second wave failed to complete the study and were thus excluded as per our preregistration plan.

All participants first completed one of the two authoritarianism scales. Democrats completed the LWA scale, whereas Republicans completed the RWA scale. Participants were then randomly assigned to read one of two stories that either violated left-wing or right-wing norms. Participants in the left-wing norm violation condition read the same story used in Studies 3 and 5 of a professor using the N-word in front of students. Participants in the right-wing norm violation condition read an adapted version of this story, wherein a professor exposes students to “transgender ideology,” which is a contemporary social issue many right-wing media outlets cover. The political valence of these two stories was pretested (Web Appendix H). Participants then completed a brief filler task. Consistent with Studies 3 and 5, participants were then offered a retributive donation option that promised to send letters to the professor's school calling for their dismissal for every donation received. Participants then rated their willingness to donate on a seven-point scale. See Web Appendix C for all stimuli and measures.

### Results

We first observed a significant main effect of ideological congruency in both samples on participants’ willingness to make a retributive donation. Specifically, Democrats were more willing to donate when they read about a left-wing norm violation (M_donation_ = 3.49) relative to a right-wing norm violation (M_donation_ = 2.07; t(397.97) = 7.37, *p *< .001), and Republicans were more willing to donate when they read about a right-wing norm violation (M_donation_ = 3.85) relative to a left-wing norm violation (M_donation_ = 1.90; t(355) = 10.08, *p *< .001).

**Figure 4.**
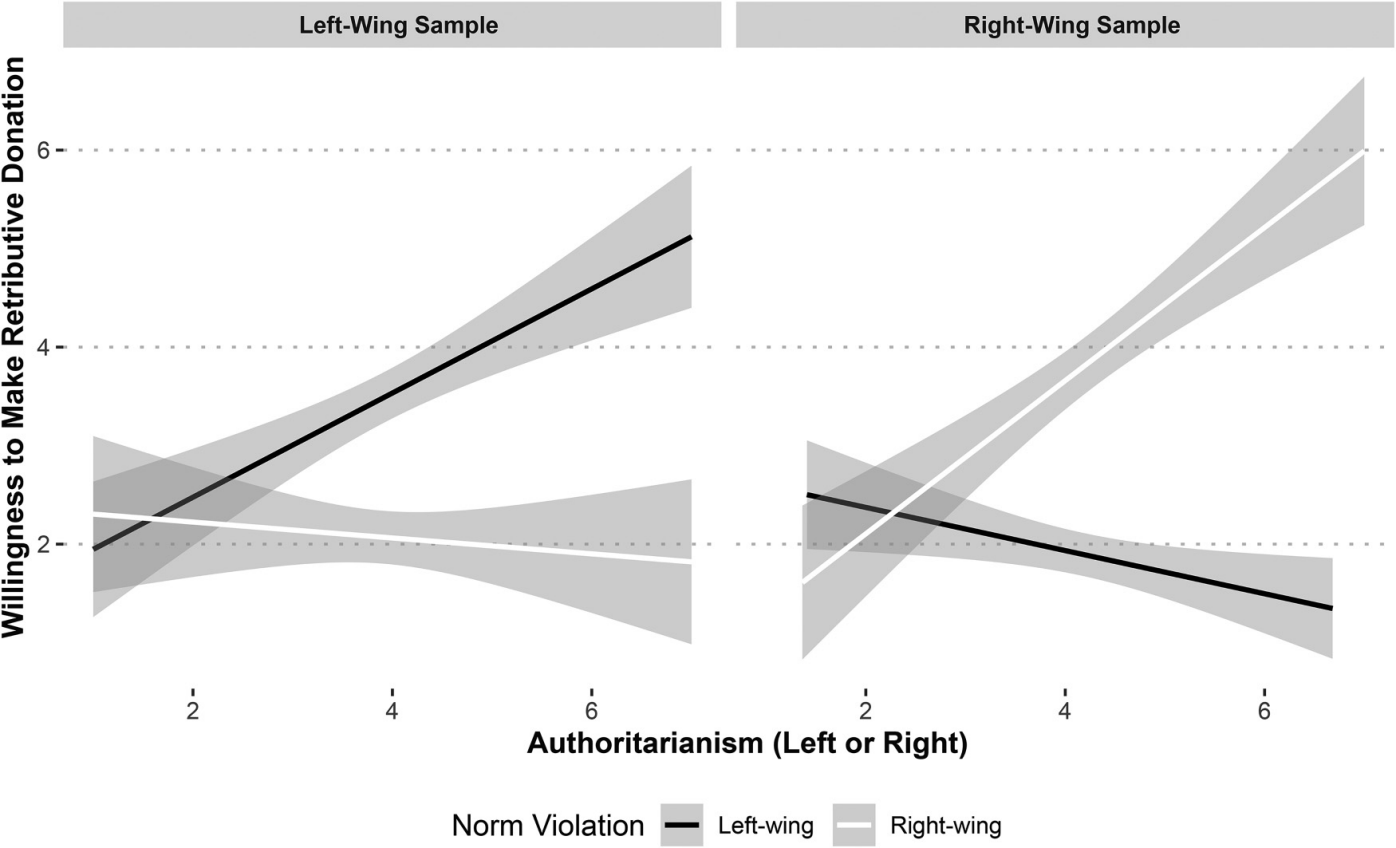
Study 6 Results.

Next, we fit two complementary linear regression models with an interaction term between each sample's relevant authoritarianism scale and our norm violation manipulation. In both models, as predicted, we observed a significant and positive interaction between authoritarianism and norm violation. Higher authoritarianism resulted in increased willingness to make retributive donations when participants were exposed to an ideologically congruent norm violation. Specifically, Democrats higher in LWA were more likely to make a retributive donation when they read about a left-wing norm violation (β = .610, SE = .171, t(396) = 3.58, *p *< .001). Republicans higher in RWA were more likely to make a retributive donation when they read about a right-wing norm violation (β = .997, SE = .158, t(390) = 6.33, *p *< .001; Figure 4).

### Discussion

The results of Study 6 indicate that authoritarianism is positively associated with prosocial behavior (H_3_) which, to our knowledge, has not been shown in the prosocial behavior literature. This theoretical distinction is important, because authoritarianism is the conceptual opposite of many traits previously found to positively predict prosocial behavior. Whereas previous work has focused on positive traits like agreeableness (Caprara, Alessandri, and Eisenberg 2012), benevolence (Caprara and Steca 2007), and empathy (Alessandri et al. 2009), we found evidence that a trait associated with disagreeableness and antagonism (Costello et al. 2022) positively influences prosocial behavior.

We conceptually replicate these results and address potential limitations of this study across several follow-up studies in our supplementary Web Appendix. In Supplementary Study 2 (Web Appendix I), we replicate a similar interaction between LWA and volitional wrongdoing but using an incentive-compatible choice. We find that as LWA increases and volitional wrongdoing is present, donors shift from making normal donations to making retributive donations. Additionally, in Supplementary Study 2 (Web Appendix I), we rule out status-seeking aggression as an alternative explanation for the influence of authoritarianism on retributive donations. In Supplementary Study 3 (Web Appendix J), we show that individuals higher in LWA form stronger negative moral judgments of volitional wrongdoers, and thus express greater intentions to make retributive donations. Finally, in both Supplementary Studies 3 (Web Appendix J) and 4 (Web Appendix K), we also find that neither reactance nor moral identity interacts with the retributive psychological processes that we explore in this work.

In summary, Study 6 focused on traits of individuals that predisposed them to making retributive donations, which again satisfies our goal of demonstrating that prosocial behaviors can be motivated by retribution. We next investigate features of appeals that are conducive to retributive donations to provide substantive insights to charitable organizations.

## Study 7: Efficacy at Punishment and Willingness to Make Retributive Donations

In Study 7, we examine a boundary condition consistent with our retribution account. Specifically, we examine the impact of punishment efficacy on retributive philanthropy. Previous work has identified efficacy (and consumers’ perceptions of efficacy) as a driver of donations (Sharma and Morwitz 2016). However, “efficacy” has traditionally been conceptualized as efficacy at helping others, often operationalized through minimization of overhead costs (Gneezy, Keenan, and Gneezy 2014). With retributive philanthropy, we propose that efficacy considerations will shift from a focus on “helping” to *punishing* others and will be conditional on consumers forming negative moral judgments of a wrongdoer. In this study, we manipulate volitional wrongdoing using stimuli from Study 3 and measure negative moral judgments. Additionally, we manipulate donation efficacy at punishing a wrongdoer. We expect that willingness to make a retributive donation will significantly increase when participants have strong negative moral judgments of volitional wrongdoing and when presented with an effective (vs. ineffective) avenue for retributive donation. Finally, our focus on efficacy enables us to demonstrate that *actual* (vs. symbolic) punishment matters to retributive donors.

### Method

Prolific Academic participants (N = 1,197; ages 18–79 years, M_age_ = 40.91 years; 50.04% female, 49.96% male) completed this study for payment and were randomly assigned to conditions in a 2 (wrongdoing: volitional vs. nonvolitional) × 2 (punishment efficacy: high vs. low) between-participants design. Our data collection and analysis plans were preregistered at https://aspredicted.org/665w-mjpg.pdf.

Participants were presented with the same volition manipulation and measures used in Study 3. Participants were then informed that a local charity was offering a retributive benefit—letters sent to the professor's dean calling for his dismissal—in exchange for donations. Efficacy was manipulated by informing participants that the fictitious dean of the university stated that (1) he does not consider public comments when making hiring or firing decisions (ineffective), or (2) the public comments are making him reconsider the professor's employment (effective). Participants then rated their likelihood of donating to the charity (1–7).

### Results

We first observed a significant difference between our volitional and nonvolitional wrongdoing conditions, such that participants expressed a greater likelihood of making a retributive donation when the professor's use of a racial slur was framed as volitional (M = 2.62) versus nonvolitional (M = 1.92; t(1,116.9) = 6.88, *p *< .001).

**Figure 5.**
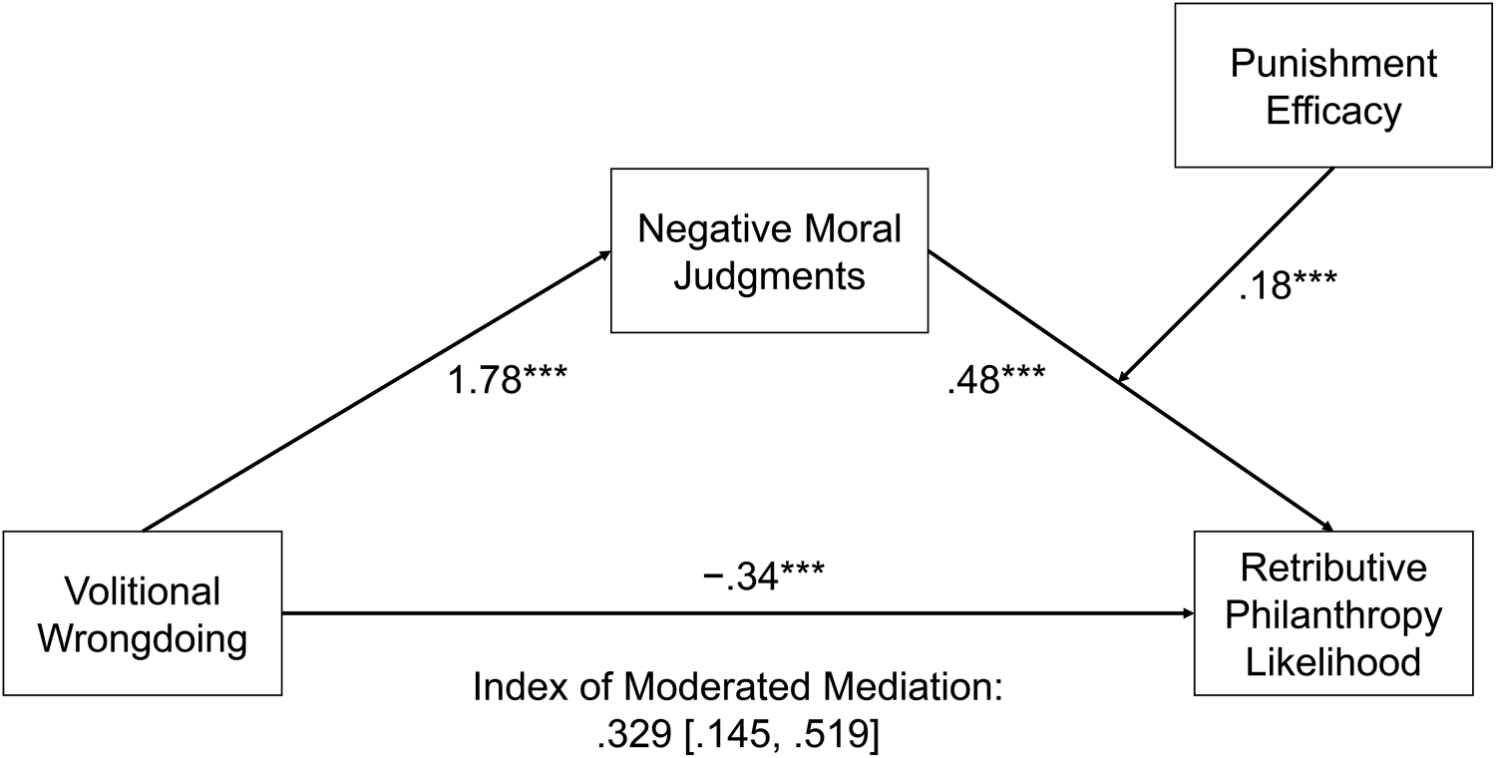
Study 7 Results.

We next used Hayes’s (2013) PROCESS Model 14 (10,000 resamples) to test the effect of volitional wrongdoing on retributive donation likelihood through negative moral judgments and conditional on punishment efficacy (Figure 5). First, we observed an overall main effect of volitional wrongdoing on negative moral judgments (β = 1.782, SE = .094, CI_95_: [1.597, 1.966], t(1,195) = 18.94, *p *< .001). Next, we examined the interactive effect of negative moral judgments (measured) and punishment efficacy on the likelihood of retributive donation. First, controlling for the effect of volitional wrongdoing, we observed direct effects of both negative moral judgment (β = .485, SE = .034, CI_95_: [.417, .552], t(1,192) = 14.16, *p *< .001) and punishment efficacy (β = −.391, SE = .168, CI_95_: [−.722, −.061], t(1,192) = −2.32, *p *= .020). Importantly, as predicted and in line with our preregistration analysis plan, we observed a significant interaction between negative moral judgments and punishment efficacy (β = .184, SE = .046, CI_95_: [.095, .274], t(1,192) = 4.04, *p *< .001). Specifically, the effect of negative moral judgments on donation was strongest when punishment efficacy was high (β = .669, SE = .035, CI_95_: [.600, .738], t(1,192) = 19.11, *p *< .001) versus low (β = .485, SE = .034, CI_95_: [.417, .552], t(1,192) = 14.16, *p *< .001). The index of moderated mediation excluded zero (index = .329, SE = .096, CI_95_: [.145, .519]), indicating that efficacy does influence willingness to make retributive donations.

### Discussion

The results of Study 7 demonstrate that if consumers believe that their retributive actions will effectively deliver punishment, they are more willing to make retributive donations, conditional on having rendered negative moral judgment of a volitional wrongdoer (H_4_). These results comport with past findings that prosocial behavior is partially driven by donors’ desire to have material impact (White, Habib, and Dahl 2019) but differs from prior theory in that the efficacy is related to punishing rather than helping. Importantly, the results of this study align with findings from our qualitative study: retributive donors want their donations to have an impact, with the desired impact being punishment (vs. helping). In addition, these results suggest that managers would be better served by proactively highlighting the efficacy of punishment when it is high or highlighting the blameworthiness of the wrongdoer instead when it is low.

The results of Study 7 demonstrate that the unique benefit of retributive philanthropy—the possibility of enacting actual, effective punishment—influences willingness to donate.

## General Discussion

Together, our 12 primary and supplementary studies employ multiple methods—qualitative, observational, and experimental—to explore the emerging phenomenon of retributive philanthropy and offer convergent evidence for its conceptual uniqueness relative to charitable giving motivated by altruism and self-interest. Studies 1 and 2 demonstrated the existence and conceptually distinct nature of retributive philanthropy as a form of donation characterized by different motives, emotions, and behaviors than self-interested or altruistic donations. Studies 3 and 4 provide strong evidence for the causal effect of volitional wrongdoing on retributive donation and its downstream impact on (and the necessity of) negative moral judgments and desire to punish. Study 5 shows that other-condemning moral emotions also play a mechanistic role in retributive donations, such that volitional wrongdoing leads to increased contempt, anger, and disgust. Studies 6 and 7 demonstrate two moderators of this process that lend support for our retribution account—authoritarian personalities and efficacy at punishing others. To summarize, we found that willingness to make retributive donations was elevated when participants (especially those with authoritarian personalities) had negative moral judgments of a volitional wrongdoer, desired to punish them, and were presented with an effective means of enacting retribution via their donation. We demonstrated these effects in a variety of contexts (e.g., antisemitism, racism, war)­ and using a variety of measures (e.g., donation likelihood, real donations, choice between charities).

### Theoretical Contributions

Our work is the first to empirically and theoretically explore how retribution can motivate donations. Our findings should be of theoretical interest to researchers, as they expand the range of situations, motives, and personalities known to influence prosocial behavior. Retributive philanthropy is distinct because it is uniquely driven by negative moral judgments, other-condemning moral emotions, and a desire to punish wrongdoers. Previous work has linked moral emotions and values with donation behaviors but has largely focused on positive emotions and values like love, compassion, and gratitude (Bartlett and DeSteno 2006; Cavanaugh, Bettman, and Luce 2015; Goenka and Van Osselaer 2019). Future work could investigate how donors reconcile their mixed motives of wanting to punish a wrongdoer and support valuable causes, as well as how reduced perceptions of personal risk interact with prior work on taboo trade-offs (Tetlock et al. 2000).

In an editorial calling for research into the “dirty underbelly” of prosocial behavior, Labroo and Goldsmith (2021) note that extant literature has principally considered altruistic and self-interested motives for prosocial behavior. Neglected, however, are interpersonal motives for prosocial behavior: How do the relationships consumers have with others influence their behavior? Prior work suggests that *positive* interpersonal interactions drive contributions (Sepehri et al. 2021) and typically in dyadic donor–beneficiary relationships or triadic donor–fundraiser–beneficiary relationships (Chapman et al. 2022). By contrast, we introduce volitional wrongdoers as a fourth agent whose *negative* relationship with a donor (evidenced by negative moral judgments and desire to punish), in conjunction with other traditional features of donor–organization relationships (e.g., efficacy perceptions), can result in increased donation behavior. Thus, in our research we answer Labroo and Goldsmith's call and start the conversation on how and why “darker” motives and relationships may lead to prosocial actions.

We also contribute to theory on the type of situations that are conducive to prosocial behavior. We demonstrate that the volition of a wrongdoer—something previously not considered in the prosocial literature—has a causal effect on willingness to make retributive donations. Future work could explore how specific types of wrongdoing alter retributive donor preferences. For example, Goenka and Van Osselaer (2019) demonstrated that matching positive emotions to positive moral foundations (e.g., compassion and care, gratitude and fairness) can lead to more and greater donations. A similar effect may be observed in the opposite direction—perhaps retributive appeals referencing purity (or lack thereof) may be more effective for converting donors experiencing disgust or other purity-related moral emotions (Wheatley and Haidt 2005).

Finally, we contribute to current theorizing on the link between efficacy and donations. Contemporary research has stressed the effect of efficacy at helping others on donations (Dai and Zhang 2019; Gneezy, Keenan, and Gneezy 2014; MacAskill 2016; Saeri et al. 2022; Sharma and Morwitz 2016; White, Habib, and Dahl 2019). By contrast, our work suggests that charitable efficacy at punishing others positively predicts retributive donations. These findings encourage a broader view of efficacy as it pertains to donation behavior. Whereas current frameworks such as SHIFT (White, Habib, and Dahl 2019) identify efficacy as related to prosocial outcomes, the integration of our work on retributive philanthropy suggests it may be better understood as “efficacy at achieving consumers’ goals.”

### Substantive Contributions and Managerial Implications

Our research offers important guidance for charitable organizations seeking to raise funds through donation. Though previous work has outlined a variety of factors and motivations leading to donations (Batson and Powell 2003; Labroo and Goldsmith 2021; White, Habib, and Dahl 2019), our work is the first to highlight retribution as a motivation for giving. In doing so, we extend the range of donation appeals charities can make. Specifically, in situations amenable to retribution—where there is a volitional wrongdoer the charity wishes to combat—retributive appeals for donations may help satisfy donors’ desires to punish the wrongdoer and result in greater funding for the charity, both by causing larger donations and attracting new donors. This may be of particular interest to charities that are currently facing novel threats to abortion rights, LGBTQ+ rights, and other civil rights. For example, in response to the overturning of *Roe v. Wade* protections for abortion in the United States, organizations like Planned Parenthood may be able to solicit further funds by offering donation benefits like sending hostile messages to Supreme Court justices or antichoice politicians. Additionally, such appeals may be more effective when the wrongdoing is framed as volitional (e.g., Studies 3, 4, 5) and the efficacy of retributive efforts (Study 7) are made salient and when a charity's target demographic is more authoritarian (Study 6).

Our findings also contribute to research on “cancel culture,” which has seen a surge in interest in academic and popular works (e.g., Lukianoff and Schlott 2023; Tosi and Warmke 2016). Indeed, some instances of retributive philanthropy could be considered manifestations of cancel culture, though the two concepts are conceptually distinct, as many would-be cancelers do not care about the degree to which wrongdoers are in control of their actions (Lukianoff and Schlott 2023). Our work nevertheless demonstrates that cancellations can be leveraged to result in greater funding for valuable social causes, particularly in cases where consumers have strong beliefs as to the volitional nature of a wrongdoing (such as in the case of Kanye West). Future research should explore how charities can best leverage cancelation events and channel cancelers’ energy to more productive ends.

Moreover, future research could seek to better understand why donation is a preferred avenue for retribution than more traditional means. We explored this question in an additional study (Web Appendix L) and reasoned that the prosocial elements of retributive philanthropy operate as a “fig leaf” that makes the aggressive elements (i.e., punishing others) less professionally, legally, and socially risky, thus leading consumers to prefer retributive donations over other means of enacting retribution.

Finally, future research can explore the long-term benefits (or costs) of charities implementing retributive appeals. Some work suggests charities with a “combative orientation” may suffer in the long run relative to less combative charities (Botner, Mishra, and Mishra 2015), and preliminary work on related phenomena like “spite philanthropy” (Witkowsky 2021) suggests that spite-oriented campaigns tend to be short-lived. Future research could therefore explore how and why the darker motives inherent to retributive philanthropy interact with more traditional self-interested or altruistic motives. Specifically, future researchers could investigate how donors rationalize wanting to both help and punish others simultaneously, and how the positive elements of retributive philanthropy may provide social or moral license for the darker elements.

In conclusion, we introduce a phenomenon that represents a significant departure from prior accounts of prosocial behavior. Prior research has historically explored how positive emotions or traits drive donation behavior. However, just as love, compassion for others, and a desire to help those in need are fundamental elements of the human experience, so too are hatred, rage at injustice, and a desire to punish wrongdoers. Addressing the most pressing issues of our time without leveraging the latter is like fighting with one hand tied behind one's back. We hope that our work helps researchers refine their models of what drives donation behavior and provides charitable organizations with more tools with which to combat injustice wherever it may be.

## Supplemental Material

sj-pdf-1-mrj-10.1177_00222437251320021 - Supplemental material for Retributive Philanthropy Supplemental material, sj-pdf-1-mrj-10.1177_00222437251320021 for Retributive Philanthropy by Ethan Milne, Kirk Kristofferson and Miranda R. Goode in Journal of Marketing Research

## Coeditor

Karen Page Winterich

## Associate Editor

Claudia Townsend

## References

[bibr1-00222437251320021] Alessandri Guido Caprara Gian Vittorio Eisenberg Nancy Steca Patrizia (2009), “Reciprocal Relations Among Self-Efficacy Beliefs and Prosociality Across Time,” Journal of Personality, 77 (4), 1229–59. 19558437 10.1111/j.1467-6494.2009.00580.x PMC2771548

[bibr2-00222437251320021] Algoe Sara B. Haidt Jonathan (2009), “Witnessing Excellence in Action: The ‘Other-Praising’ Emotions of Elevation, Gratitude, and Admiration,” Journal of Positive Psychology, 4 (2), 105–27. 19495425 10.1080/17439760802650519 PMC2689844

[bibr3-00222437251320021] Altemeyer Bob (2007), The Authoritarians. Manitoba University Press.

[bibr4-00222437251320021] Ames Daniel L. Fiske Susan T. (2013), “Intentional Harms Are Worse, Even When They’re Not,” Psychological Science, 24 (9), 1755–62. 23878021 10.1177/0956797613480507 PMC4470288

[bibr5-00222437251320021] Antonetti Paolo (2016), “Consumer Anger: A Label in Search of Meaning,” European Journal of Marketing, 50 (September), 1602–28.

[bibr6-00222437251320021] Bartlett Monica Y. DeSteno David (2006), “Gratitude and Prosocial Behavior: Helping When It Costs You,” Psychological Science, 17 (4), 319–25. 16623689 10.1111/j.1467-9280.2006.01705.x

[bibr7-00222437251320021] Batson Daniel (2022), “Prosocial Motivation: A Lewinian Approach,” Motivation Science, 8, 1–10.

[bibr8-00222437251320021] Batson Daniel Powell Adam A. (2003), “Altruism and Prosocial Behavior,” in Handbook of Psychology: Personality and Social Psychology, Vol. 5, Theodore Millon, Melvin J. Lerner, and Irving B. Weiner, eds. John Wiley & Sons, Inc, 463–84.

[bibr9-00222437251320021] Botner Keith A. Mishra Arul Mishra Himanshu (2015), “What’s in a Message? The Longitudinal Influence of a Supportive Versus Combative Orientation on the Performance of Nonprofits,” Journal of Marketing Research, 52 (1), 39–55.

[bibr10-00222437251320021] Brady William J. McLoughlin Killian Doan Tuan N. Crockett Molly J. (2021), “How Social Learning Amplifies Moral Outrage Expression in Online Social Networks,” Science Advances, 7 (33), eabe5641. 10.1126/sciadv.abe5641 PMC8363141 34389534

[bibr11-00222437251320021] Caprara Gian Vittorio Alessandri Guido Eisenberg Nancy (2012), “Prosociality: The Contribution of Traits, Values, and Self-Efficacy Beliefs,” Journal of Personality and Social Psychology, 102 (6), 1289–303. 21942280 10.1037/a0025626

[bibr12-00222437251320021] Caprara Gian Vittorio Steca Patrizia (2007), “Prosocial Agency: The Contribution of Values and Self–Efficacy Beliefs to Prosocial Behavior Across Ages,” Journal of Social and Clinical Psychology, 26 (2), 218–39.

[bibr13-00222437251320021] Cavanaugh Lisa A. Bettman James R. Luce Mary Frances (2015), “Feeling Love and Doing More for Distant Others: Specific Positive Emotions Differentially Affect Prosocial Consumption,” Journal of Marketing Research, 52 (5), 657–73.

[bibr14-00222437251320021] Chapman Cassandra M. Louis Winnifred R. Masser Barbara M. Thomas Emma F. (2022), “Charitable Triad Theory: How Donors, Beneficiaries, and Fundraisers Influence Charitable Giving,” Psychology & Marketing, 39 (9), 1826–48.

[bibr15-00222437251320021] Cherry Myisha (2021), The Case for Rage. Oxford University Press.

[bibr16-00222437251320021] Costello Thomas H. Bowes Shauna M. Stevens Sean T. Waldman Irwin D. Tasimi Arber Lilienfeld Scott O. (2022), “Clarifying the Structure and Nature of Left-Wing Authoritarianism,” Journal of Personality and Social Psychology, 122 (1), 135–70. 34383522 10.1037/pspp0000341

[bibr17-00222437251320021] Cryder Cynthia E. Loewenstein George Seltman Howard (2013), “Goal Gradient in Helping Behavior,” Journal of Experimental Social Psychology, 49 (6), 1078–83.

[bibr18-00222437251320021] Curry Oliver Scott Rowland Lee A. Van Lissa Caspar J. Zlotowitz Sally McAlaney John Whitehouse Harvey (2018), “Happy to Help? A Systematic Review and Meta-Analysis of the Effects of Performing Acts of Kindness on the Well-Being of the Actor,” Journal of Experimental Social Psychology, 76, 320–29.

[bibr19-00222437251320021] Dai Hengchen Zhang Dennis J. (2019), “Prosocial Goal Pursuit in Crowdfunding: Evidence from Kickstarter,” Journal of Marketing Research, 56 (3), 498–517.

[bibr20-00222437251320021] Djan Edward (2023), “Think Your Ex Was a Cockroach? Toronto Zoo’s New Name-a-Roach Campaign Will Let You Make It Official,” The Toronto Star (January 16), https://www.thestar.com/amp/news/gta/2023/01/16/think-your-ex-was-a-cockroach-toronto-zoos-new-name-a-roach-campaign-will-let-you-make-it-official.html.

[bibr21-00222437251320021] Feather Norman T. (2005), Values, Achievement, and Justice: Studies in the Psychology of Deservingness. Springer. 10.1207/s15327957pspr0302_1 15647142

[bibr22-00222437251320021] Finkel Norman J. Slobogin Christopher (1995), “Insanity, Justification, and Culpability Toward a Unifying Schema,” Law and Human Behavior, 19 (5), 447–64.

[bibr23-00222437251320021] Fitness Julie (2001), “Betrayal, Rejection, Revenge, and Forgiveness,” in Interpersonal Rejection, Mark R. Leary, ed. Oxford University Press, 73–103.

[bibr24-00222437251320021] Flaherty Colleen (2020), “Failure to Communicate,” Inside Higher Ed (September 8), https://www.insidehighered.com/news/2020/09/08/professor-suspended-saying-chinese-word-sounds-english-slur.

[bibr25-00222437251320021] Glazek Christopher (2013), “The Story Behind ‘Fitch the Homeless,’” The New Yorker (June 19), https://www.newyorker.com/culture/culture-desk/the-story-behind-fitch-the-homeless.

[bibr26-00222437251320021] Gneezy Uri Keenan Elizabeth A. Gneezy Ayelet (2014), “Avoiding Overhead Aversion in Charity,” Science, 346 (6209), 632–35. 25359974 10.1126/science.1253932

[bibr27-00222437251320021] Goenka Shreyans van Osselaer Stijn M.J. (2019), “Charities Can Increase the Effectiveness of Donation Appeals by Using a Morally Congruent Positive Emotion,” Journal of Consumer Research, 46 (4), 774–90.

[bibr28-00222437251320021] Gold Natalie Pulford Briony D. Colman Andrew M. (2015), “Do as I Say, Don’t Do as I Do: Differences in Moral Judgments Do Not Translate into Differences in Decisions in Real-Life Trolley Problems,” Journal of Economic Psychology, 47, 50–61.

[bibr29-00222437251320021] Grégoire Yany Laufer Daniel Tripp Thomas (2010), “A Comprehensive Model of Customer Direct and Indirect Revenge: Understanding the Effects of Perceived Greed and Customer Power,” Journal of the Academy of Marketing Science, 38, 738–58.

[bibr30-00222437251320021] Griffin Louise (2019), “Hbomberguy Hits Back at Graham Linehan after Mermaids Charity Stream,” *Metro* (January 21), https://metro.co.uk/2019/01/21/hbomberguy-hits-back-at-graham-linehan-after-mermaids-charity-stream-every-time-you-tweet-five-people-donate-8371438/.

[bibr31-00222437251320021] Grubbs Joshua B. Warmke Brandon Tosi Justin James A. Shanti Campbell W. Keith (2019), “Moral Grandstanding in Public Discourse: Status-Seeking Motives as a Potential Explanatory Mechanism in Predicting Conflict,” PLOS One, 14 (10), e0223749. 10.1371/journal.pone.0223749 PMC6795490 31618235

[bibr32-00222437251320021] Haidt J. (2001), “The Emotional Dog and Its Rational Tail: A Social Intuitionist Approach to Moral Judgment,” Psychological Review, 108 (4), 814–34. 11699120 10.1037/0033-295x.108.4.814

[bibr33-00222437251320021] Hayes Andrew F. (2013), Introduction to Mediation, Moderation, and Conditional Process Analysis. Introduction to Mediation, Moderation, and Conditional Process Analysis: A Regression-Based Approach. Guilford Press.

[bibr34-00222437251320021] Hui Bryant P.H. Ng Jacky C.K. Berzaghi Erica Cunningham-Amos Lauren A. Kogan Aleksandr (2020), “Rewards of Kindness? A Meta-Analysis of the Link Between Prosociality and Well-Being,” Psychological Bulletin, 146 (12), 1084–1116. 32881540 10.1037/bul0000298

[bibr35-00222437251320021] Jackson Joshua Conrad Choi Virginia K. Gelfand Michele J. (2019), “Revenge: A Multilevel Review and Synthesis,” Annual Review of Psychology, 70 (1), 319–45. 10.1146/annurev-psych-010418-103305 30609911

[bibr36-00222437251320021] Jankowicz Mia (2022), “Ukrainian Soldiers Raise Money by Writing Custom Notes on Artillery Shells for $40 Before Firing Them at Russians,” Business Insider (June 16), https://www.businessinsider.com/ukraine-soldiers-custom-notes-artillery-shells-40-dollars-fire-russia-2022-6.

[bibr37-00222437251320021] Kähr Andrea Nyffenegger Bettina Krohmer Harley Hoyer Wayne D. (2016), “When Hostile Consumers Wreak Havoc on Your Brand: The Phenomenon of Consumer Brand Sabotage,” Journal of Marketing, 80 (3), 25–41.

[bibr38-00222437251320021] Karppinen Helena King Olivia Russell Pascale Sophie (2023), “Hostile Emotions and Close Relationships: Anger Can Be Related to Constructive Responses,” Personality and Individual Differences, 212, 112258.

[bibr39-00222437251320021] Klonick Kate (2016), “Re-Shaming the Debate: Social Norms, Shame, and Regulation in an Internet Age,” Maryland Law Review, 75 (4), 1029.

[bibr40-00222437251320021] Kristofferson Kirk White Katherine Peloza John (2014), “The Nature of Slacktivism: How the Social Observability of an Initial Act of Token Support Affects Subsequent Prosocial Action,” Journal of Consumer Research, 40 (6), 1149–66.

[bibr41-00222437251320021] Labroo Aparna A. Goldsmith Kelly (2021), “The Dirty Underbelly of Prosocial Behavior: Reconceptualizing Greater Good as an Ecosystem with Unintended Consequences,” Journal of Consumer Psychology, 31 (3), 417–28.

[bibr42-00222437251320021] Latifi Fortesa (2022), “Teen Activist Attacked By Rep. Gaetz Raises Massive Sums for Abortion Rights,” Teen Vogue (July 27), https://www.teenvogue.com/story/matt-gaetz-teen-abortion-donation-fundraiser.

[bibr43-00222437251320021] Levitz Eric (2022), “Kanye West Can Be Bipolar and Accountable for His Antisemitism,” Intelligencer (October 12), https://nymag.com/intelligencer/2022/10/kanye-west-anti-semitism-mental-illness-bipolar-jews-white-lives-matter.html.

[bibr44-00222437251320021] Lindenmeier Jörg Schleer Christoph Pricl Denise (2012), “Consumer Outrage: Emotional Reactions to Unethical Corporate Behavior,” Journal of Business Research, 65 (9), 1364–73.

[bibr45-00222437251320021] Lukianoff Greg Schlott Rikki (2023), The Canceling of the American Mind. Simon & Schuster.

[bibr46-00222437251320021] MacAskill William (2016), Doing Good Better. Penguin Random House.

[bibr47-00222437251320021] MacKinnon Kinnon Ross Kia Hannah Lacombe-Duncan Ashley (2021), “Examining TikTok’s Potential for Community-Engaged Digital Knowledge Mobilization with Equity-Seeking Groups,” Journal of Medical Internet Research, 23 (12), e30315. 10.2196/30315 PMC8704107 34889739

[bibr48-00222437251320021] Malle Bertram F. (2021), “Moral Judgments,” Annual Review of Psychology, 72 (1), 293–318. 10.1146/annurev-psych-072220-104358 32886588

[bibr49-00222437251320021] Marwick Alice E. (2021), “Morally Motivated Networked Harassment as Normative Reinforcement,” Social Media + Society, 7 (2), 20563051211021378.

[bibr50-00222437251320021] McCracken Grant David (1988), The Long Interview. Sage.

[bibr51-00222437251320021] McDade Aaron (2022), “GiveSendGo Says It Will Refund Remaining Donations to Truckers Convoy,” Newsweek (March 10), https://www.newsweek.com/givesendgo-says-it-will-refund-remaining-donations-truckers-convoy-1686992.

[bibr52-00222437251320021] Mercier Brett Norris Adam Shariff Azim F. (2018), “Muslim Mass Shooters Are Perceived as Less Mentally Ill and More Motivated by Religion,” Psychology of Violence, 8 (6), 772–81.

[bibr53-00222437251320021] Mettler Katie (2016), “People Are Donating to Planned Parenthood in Mike Pence’s Name,” Washington Post (November 15), https://www.washingtonpost.com/news/morning-mix/wp/2016/11/15/people-are-donating-to-planned-parenthood-in-mike-pences-name/.

[bibr54-00222437251320021] Miles Matthew B. Huberman A.M. Saldaña Johnny (2014), Qualitative Data Analysis: A Methods Sourcebook, 3rd ed. Sage.

[bibr55-00222437251320021] Peloza John Steel Piers (2005), “The Price Elasticities of Charitable Contributions: A Meta-Analysis,” Journal of Public Policy & Marketing, 24 (2), 260–72.

[bibr56-00222437251320021] Preston Caroline (2017), “With Anti-Trump Windfall, Nonprofits Are Digging In for a Fight,” The Chronicle of Philanthropy (January 31), https://www.philanthropy.com/article/with-anti-trump-windfall-nonprofits-are-digging-in-for-a-fight/.

[bibr57-00222437251320021] Przepiorka Wojtek Berger Joël (2016), “The Sanctioning Dilemma: A Quasi-Experiment on Social Norm Enforcement in the Train,” European Sociological Review, 32 (3), 439–51.

[bibr58-00222437251320021] Ryan Lisa (2016), “Here’s How Many Planned Parenthood Donations Have Been Made in Mike Pence’s Name,” The Cut (December 8), https://www.thecut.com/2016/12/how-many-planned-parenthood-donations-came-from-mike-pence.html.

[bibr59-00222437251320021] Saeri Alexander K. Slattery Peter Lee Joannie Houlden Thomas Farr Neil Gelber Romy L. Stone Jake Stone Jake Huuskes Lee Timmons Shane Windle Kai Spajic Luke Freeman Luke Moss David Behar Jon Schubert Stefan Grundy Emily A.C. Zorker Michael (2022), “What Works to Increase Charitable Donations? A Meta-Review with Meta-Meta-Analysis,” VOLUNTAS: International Journal of Voluntary and Nonprofit Organizations, 34, 626–42.

[bibr60-00222437251320021] Saleem Muniba Barlett Christopher P. Anderson Craig A. Hawkins Ian (2017), “Helping and Hurting Others: Person and Situation Effects on Aggressive and Prosocial Behavior as Assessed by the Tangram Task,” Aggressive Behavior, 43 (2), 133–46. 27629104 10.1002/ab.21669

[bibr61-00222437251320021] Schnurr Benedikt Fuchs Christoph Maira Elisa Puntoni Stefano Schreier Martin van Osselaer Stijn M.J. (2022), “Sales and Self: The Noneconomic Value of Selling the Fruits of One’s Labor,” Journal of Marketing, 86 (3), 40–58.

[bibr62-00222437251320021] Sepehri Amir Duclos Rod Kristofferson Kirk Vinoo Poornima Elahi Hamid (2021), “The Power of Indirect Appeals in Peer-to-Peer Fundraising: Why ‘S/He’ Can Raise More Money for Me Than ‘I’ Can For Myself,” Journal of Consumer Psychology, 31 (3), 612–20.

[bibr63-00222437251320021] Sharma Eesha Morwitz Vicki G. (2016), “Saving the Masses: The Impact of Perceived Efficacy on Charitable Giving to Single vs. Multiple Beneficiaries,” Organizational Behavior and Human Decision Processes, 135, 45–54.

[bibr64-00222437251320021] Small Deborah Cryder Cynthia (2016), “Prosocial Consumer Behavior,” Current Opinion in Psychology, 10, 107–11.

[bibr65-00222437251320021] Sommerland Joe (2019), “Woman Trolls Anti-Vaxxers by Donating Money for Vaccines in Their Name,” *The Independent* (July 8), https://www.indy100.com/celebrities/antivaxxers-zoe-quinn-vaccine-donations-trolling-diseases-health-8825206.

[bibr66-00222437251320021] Sommers Tamler (2016), “The Three Rs: Retribution, Revenge, and Reparation,” Philosophia, 44 (2), 327–42.

[bibr67-00222437251320021] Sommers Tamler (2022), “Metaskepticism,” in The Oxford Handbook of Moral Responsibility, Dana Kay Nelkin, ed. Oxford University Press.

[bibr68-00222437251320021] Spiggle Susan. (1994), “Analysis and Interpretation of Qualitative Data in Consumer Research,” Journal of Consumer Research, 21 (3), 491–503.

[bibr69-00222437251320021] Taylor Jennifer A. Miller-Stevens Katrina (2022), Rage Giving, 1st ed. Cambridge University Press.

[bibr70-00222437251320021] Tetlock Philip E. (2002), “Social Functionalist Frameworks for Judgment and Choice: Intuitive Politicians, Theologians, and Prosecutors,” Psychological Review, 109 (3), 451–71. 12088240 10.1037/0033-295x.109.3.451

[bibr71-00222437251320021] Tetlock Philip E. Kristel Orie V. Elson S. Beth Green Melanie C. Lerner Jennifer S. (2000), “The Psychology of the Unthinkable: Taboo Trade-Offs, Forbidden Base Rates, and Heretical Counterfactuals,” Journal of Personality and Social Psychology, 78 (5), 853–70. 10821194 10.1037//0022-3514.78.5.853

[bibr72-00222437251320021] Tosi Justin Warmke Brandon (2016), “Moral Grandstanding,” Philosophy and Public Affairs, 44 (3), 197–217.

[bibr73-00222437251320021] Van Doorn Janne Zeelenberg Marcel Breugelmans Seger M. (2014), “Anger and Prosocial Behavior,” Emotion Review, 6 (3), 261–68.

[bibr74-00222437251320021] Wheatley Thalia Haidt Jonathan (2005), “Hypnotic Disgust Makes Moral Judgments More Severe,” Psychological Science, 16 (10), 780–84. 16181440 10.1111/j.1467-9280.2005.01614.x

[bibr75-00222437251320021] White Katherine Argo Jennifer (2009), “Social Identity Threat and Consumer Preferences,” Journal of Consumer Psychology, 19, 313–25.

[bibr76-00222437251320021] White Katherine Habib Rishad Dahl Darren W. (2019), “A Review and Framework for Thinking About the Drivers of Prosocial Consumer Behavior,” Journal of the Association for Consumer Research, 5 (1), 2–18.

[bibr77-00222437251320021] Whitley Sarah C. Garcia-Rada Ximena Bardhi Fleura Ariely Dan Morewedge Carey K. (2022), “Relational Spending in Funerals: Caring for Others Loved and Lost,” Journal of Consumer Psychology, 32 (2), 211–31.

[bibr78-00222437251320021] Williams Bernard (1981), Moral Luck: Philosophical Papers 1973–1980, reissue ed. Cambridge University Press.

[bibr79-00222437251320021] Witkowsky George (2021), “Move Over, Rage Philanthropy. It’s Time for Spite Philanthropy,” Chronical of Philanthropy (April), https://www.philanthropy.com/article/move-over-rage-philanthropy-its-time-for-spite-philanthropy.

