# Supplementary material for: Retributive Philanthropy
Source: J Mark Res. 2025 Feb 6;62(5):918–36. doi: 10.1177/00222437251320021 (PMC13046273; doi:10.1177/00222437251320021)
Supplement: sj-pdf-1-mrj-10.1177_00222437251320021 - Supplemental material for Retributive Philanthropy [file sj-pdf-1-mrj-10.1177_00222437251320021.pdf]

RETRIBUTIVE PHILANTHROPY

Ethan Milne (emilne.phd@ivey.ca)
Kirk Kristofferson (kkristofferson@ivey.ca)
Miranda Goode (mgoode@ivey.ca)

Web Appendix[1]

- Web Appendix A: Overview of Web Appendix Studies……………………………..2
- Web Appendix B: Qualitative Interview Recruitment and Guide……………………3
- Web Appendix C: Stimuli and Measures (All Studies)………………………………4
- Web Appendix D: Post-Test Study 1……………………………………..………30
- Web Appendix E: Post-Test Study 2…………………………………………………...31
- Web Appendix F: Supplementary Study 1……………………..…….…………...32
- Web Appendix G: Study 5 Additional Analysis…..………………………………...35
- Web Appendix H: Post-Test Study 3……………………………….…...………..37
- Web Appendix I: Supplementary Study 2……………..………..…………………..…39
- Web Appendix J: Supplementary Study 3………………..……………………..…..…43
- Web Appendix K: Supplementary Study 4………………..……………….…….....48
- Web Appendix L: Supplementary Study 5……………..………………..…...…....52

[1] Disclaimer: These materials have been supplied by the authors to aid in the understanding of their paper. The AMA is sharing these materials at the request of the authors.

Table W1: Overview of Web Appendix Studies

**Supplementary Study 1**<br>*Study 4 Follow-Up*	▪ **Design:** 2 (Wrongdoing: volitional vs. non-volitional)<br>▪ **DV:** Likelihood of Retributive Donation<br>▪ **Focal Test:** Mediation with Negative Moral Judgments and Desire to Punish (Pre-registered)<br>▪ **Focal result:** index of serial mediation = .044, SE = 0.009, 95% CI: .028, .061	
	Volitional	Non-volitional
Donation Likelihood	3.21	2.93
**Supplementary Study 2**<br>*Authoritarianism Follow-Up Study*	▪ **Design:** 2 (Wrongdoing: volitional vs. non-volitional) x continuous (Left-Wing Authoritarianism)<br>▪ **DV:** Choice of Retributive Charity<br>▪ **Focal Test:** Multinomial logistic regression on choice between charities and not donating<br>▪ **Focal result:** Volitional wrongdoing x LWA = 0.84, 95% CI: 0.15, 1.52, p = .017	
	Volitional	Non-volitional
Retributive Charity Choice	27.03%	16.39%
**Supplementary Study 3**<br>*Personality Follow-up Study*	▪ **Design:** 2 (Wrongdoing: volitional vs. non-volitional) x Continuous (LWA, Reactance, Moral Identity)<br>▪ **DV:** Likelihood of Retributive Donation<br>▪ **Focal Test:** Mediation with Negative Moral Judgments (Pre-registered)<br>▪ **Focal result:** index of moderated mediation (LWA) = .072, SE = .029, 95% CI: .016, .128	
	Volitional	Non-volitional
Donation Likelihood	2.99	2.44
**Supplementary Study 4**<br>Reactance and Moral Identity Follow-Up Study	▪ **Design:** 2 (Wrongdoing: volitional vs. non-volitional) x Continuous (Reactance, Moral Identity)<br>▪ **DV:** Likelihood of Retributive Donation<br>▪ **Focal Test:** Mediation with Negative Moral Judgments (Pre-registered)<br>▪ **Focal result:** Index of mediation = 0.138, SE = .043, 95% CI: .059, .228	
	Charity	For-Profit
Donation Likelihood	3.36	3.35
**Supplementary Study 5**<br>*Retributive Donation vs. For-Profit Payment for Retribution*	▪ **Design:** 2 (Retributive option: for-profit payment vs. non-profit donation)<br>▪ **DV:** Likelihood of Retributive Donation<br>▪ **Focal Test:** Mediation with Perceived Personal Risk (Pre-registered)<br>▪ **Focal result:** index of mediation = .161, SE = .043, 95% CI: .102, .572	
	Volitional	Non-volitional
Donation Likelihood	2.36	1.86

Web Appendix B: Qualitative Interview Recruitment and Guide

Recruitment

The recruitment message was as follows:

"Are you interested in participating in a real scientific study? We're currently recruiting participants on TikTok for a study that we plan to run over the next couple of months. We're studying a phenomenon we call retributive philanthropy, or when people make donations in order to punish someone else. An example might be when people donated to Planned Parenthood in Mike Pence's name to get back at him for his opposition to abortion rights, or if a teen donates to the Trevor Project to get back at parents who don't accept their sexuality. If you've made a donation like this and you're interested in sharing your experience, please go to the link in my bio and that will take you to a google form with some more information."

Interview Guide

The interview guide used for this qualitative study was as follows:

1. Please tell me a bit about yourself
   - What region do you live in?
   - What's your educational background?
   - What's been your work history?
2. Please tell me about your general experiences with giving to charity
   - Do you donate to charity often?
   - When you donate, do you donate small amounts or large amounts?
   - What generally motivates you to give to charity?
   - How do you choose which charities to donate to?
   - How does donating to charity typically make you feel?
3. We specifically recruited participants who have had experience making what might be called a "retributive donation." Could you tell me about that experience?
   - What's the story behind you making that donation?
   - What motivated you to make that donation?
   - How did it feel to make that donation?
   - Would you make a similar donation in the future?
   - Did you donate more or less than you usually do?
   - Would you describe yourself as a petty or vengeful person?
4. Some charities are interested in attracting people who make retributive donations. Do you think this is a good idea for them to do?

Web Appendix C: Stimuli and Measures (All Studies)

Study 3

Stimuli

Volitional Wrongdoing Present Condition

A professor at the University of Western Kentucky is currently being protested by several student groups for intentionally saying an anti-Black slur.

Late last month, John Gerber, the professor, was teaching a lesson on “friendly words” in English – think “pal,” “buddy” or “homie” in English – in his master’s level course on communication for management.

“Using friendly words can help bring the audience in,” Gerber said, according to a recording of one of the Zoom course sections and a transcription that appeared next to him on screen. Gerber then said the N-word as an example of a friendly word used in Black communities.

Gerber, who has worked in Black communities but is not himself Black, did not warn students that he would be saying the N-word. And some or all of the Black students across three sections of the course were offended by what they’d heard. So they wrote a letter to the dean of the Johnston School of Business, Kyle Karminsky, among others, describing Gerber as insensitive and incapable of teaching the three week intensive communications course.

“What we heard in class was a hurtful word with tremendous implications for the Black community,” wrote the students, who identified themselves as Black MBA Candidates c/o 2022. “There are many different ways to communicate that this word is used in Black communities, but Professor Gerber’s decision to say the word is hurtful and unacceptable to our University of Western Kentucky community. The negligence and disregard displayed by our professor was very clear in today’s class.”

The students said some of them had voiced their concern to Gerber during his lecture, but that he’d deliberately used the word in following class sections anyway. They also said he justified his use of the anti-Black slur by claiming that “the purpose of university is to make you uncomfortable”, and that he “hoped

[his] students are made uncomfortable in every class, because that's the only way we can grow as communicators."

Volitional Wrongdoing Absent Condition

A professor at the University of Western Kentucky is currently being protested by several student groups for saying a Chinese word that sounds like an anti-black slur.

Late last month, John Gerber, the professor, was teaching a lesson on "filler words" in other languages – think "err," "um" or "like" in English – in his master's-level course on communication for management.

"Taking a break between ideas can help bring the audience in," Gerber said, according to a recording of one of the Zoom course sections and a transcription that appeared next to him on screen. "In China," for instance, he continued, "the common pause word is 'that that that.' So in China it might be ne ga, ne ga, ne ga."

Gerber, who has worked in China but is not a scholar of Chinese, did not warn students that ne ga, (alternatively spelled nà ge, or neige) sounds something like the N-word – which it does. And some or all of the Black students across three sections of the course were offended by what they'd heard. So they wrote a letter to the dean of the Johnston School of Business, Kyle Karminsky, among others, describing Gerber as insensitive and incapable of teaching the three-week intensive communications course.

"The way we heard it in class was indicative of a much more hurtful word with tremendous implications for the Black community," wrote the students, who identified themselves as Black MBA Candidates c/o 2022. "There are over 10,000 characters in the Chinese written language and to use this phrase, a clear synonym with this derogatory N-Word term, is hurtful and unacceptable to our University of Western Kentucky community. The negligence and disregard displayed by our professor was very clear in today's class."

The students said some of them had voiced their concern to Gerber during his lecture, but that he'd used the word in following class sections anyway with proper pronunciation. Fellow Chinese students "confirmed that Professor Gerber properly pronounced the word in class."

Measures

DV

Thank you for taking the time to answer these questions. Given the nature of this study, you will be awarded an additional $1 as bonus compensation for your time.

Retributive Option Present Condition:

Participants were shown the following options:

> You can either **keep this bonus for yourself.**
>
> OR **donate it** to one of two Kentucky non-profits raising funds in response to the situation you read about in this study.
>
> The **Kentucky Antiracist Students Alliance** promises to use all funds raised to run a series of anti-racist workshops for students and faculty. For every donation, the organization also promises to send a letter to the university calling for Professor Gerber's dismissal.
>
> The **Western Kentucky Black Students Group** promises to use all funds raised to run a series of anti-racist workshops for students and faculty.

Participants then selected one of three options presented in a horizontal list (order randomized): 1) Keep my bonus, 2) donate to the Kentucky Antiracist Students Alliance, or 3) donate to the Western Kentucky Black Students Group. Participants who selected "keep my donation" were given a $1 coin by a supervising research assistant.

Retributive Option Absent Condition:

Participants were shown the following options:

> You can either **keep this bonus for yourself.**
>
> OR **donate it** to one of two Kentucky non-profits raising funds in response to the situation you read about in this study.
>
> The **Kentucky Antiracist Students Alliance** promises to use all funds raised to run a series of anti-racist workshops for students and faculty.

> The **Western Kentucky Black Students Group** promises to use all funds raised to run a series of anti-racist workshops for students and faculty.

Participants then selected one of three options presented in a horizontal list (order randomized): 1) Keep my bonus, 2) donate to the Kentucky Antiracist Students Alliance, or 3) donate to the Western Kentucky Black Students Group. Participants who selected "keep my donation" were given a $1 coin by a supervising research assistant.

Study 4

Stimuli

Volitional Wrongdoing Present Condition

No, Ye's mental health does not excuse antisemitism, experts say

It's hard to ignore Ye's string of controversies. In the past few days, the critically acclaimed rapper has been making headlines for wearing a "White Lives Matter" T-shirt at Paris Fashion Week, bullying Vogue fashion editor Gabriella Karefa Johnson, and, most recently, sharing antisemitic statements before being locked out of his social media accounts.

The rapper, also known by his given name Kanye West, has opened up about his bipolar disorder diagnosis, though we don't know what role it is playing in his recent behaviors. In some cases, it's true that suffering from a manic episode can influence "aberrant behavior" that isn't indicative of one's general moral values. On the flip side, it can also expose suppressed personal beliefs.

"We want to recognize that this person may have their own very strong beliefs on religion or politics, and we want to call that out as being separate from the mental health diagnosis," says Carla Manly, a clinical psychologist and author of "Joy from Fear."

"There are many people who don't have mental health issues who are racist and bigoted. And there are people with mental health issues who are not racist or bigoted. We want to see those as two very different issues." In short, mental illness does not excuse racism, bigotry or misogyny.

Psychologists say unmanaged mental health conditions can cause people to act in seemingly uncharacteristic ways. But mental illness and bigotry are two distinct problems requiring two distinct solutions.

Whether or not he is suffering from an episode, his words cause irreparable harm. The Anti-Defamation League, which tracks antisemitic behavior nationwide, reported 2,717 incidents in 2021 – a 34% increase from the year prior. Celebrities like Jamie Lee Curtis and Sarah Silverman have condemned Ye for his hateful message.

"The holiest day in Judaism was last week. Words matter. A threat to Jewish people ended once in a genocide. Your words hurt and incite violence. You are a father. Please stop," Curtis tweeted Sunday.

We should hold people accountable for their bad behavior.

With high-profile stars like Ye, whose words have a profound influence on his young, impressionable fans, hateful rhetoric can be particularly damaging. But as spectators who don't know him personally, the best thing we can do is to stop rewarding his problematic behavior with attention.

This doesn't mean ignoring the severity of Ye's conduct. Rather "not sharing his messages, not endorsing them, not repeating them – and rejecting them firmly with the gentle suggestion (especially by those closest to him) that he is not his best self right now and could use some kind of support to move away from this toxic behavior," she suggests.

Volitional Wrongdoing Absent Condition

<u>Ye's mental health likely caused antisemitism, experts say</u>

It's hard to ignore Ye's string of controversies. In the past few days, the critically acclaimed rapper has been making headlines for wearing a "White Lives Matter" T-shirt at Paris Fashion Week, bullying Vogue fashion editor Gabriella Karefa Johnson, and, most recently, sharing antisemitic statements before being locked out of his social media accounts.

The rapper, also known by his given name Kanye West, has opened up about his bipolar disorder diagnosis, which may play a role in his recent behaviors. Suffering from a manic episode can often influence "aberrant behavior" that isn't indicative of one's general moral values.

"We want to recognize that this person may have their own very strong beliefs on religion or politics, and that their mental health may lead them to acting in ways contrary to those beliefs during a manic episode," says Carla Manly, a clinical psychologist and author of "Joy from Fear."

"There are many people who don't have mental health issues who are racist and bigoted. And there are people who are not racist or bigoted but experiencing mental health issues that make them act badly. We want to see those as two very different issues." In short, mental illness could cause racism, bigotry, or misogyny.

Psychologists say unmanaged mental health conditions can cause people to act in seemingly uncharacteristic ways, which lead them to behave in a seemingly bigoted manner.

Whether or not he is suffering from an episode, his words cause irreparable harm. The Anti-Defamation League, which tracks antisemitic behavior nationwide, reported 2,717 incidents in 2021 – a 34% increase from the year prior. Celebrities

like Jamie Lee Curtis and Sarah Silverman have condemned Ye for his hateful message.

"The holiest day in Judaism was last week. Words matter. A threat to Jewish people ended once in a genocide. Your words hurt and incite violence. You are a father. Please stop," Curtis tweeted Sunday.

We should show compassion while helping those suffering mental illness get the treatment they need.

With high-profile stars like Ye, whose words have a profound influence on his young, impressionable fans, hateful rhetoric can be "particularly damaging," Bonior warns. But as spectators who don't know him personally, the best thing we can do is to stop rewarding his problematic behavior with attention.

This doesn't mean ignoring the severity of Ye's conduct. Rather "not sharing his messages, not endorsing them, not repeating them – and rejecting them firmly with the gentle suggestion (especially by those closest to him) that he is not his best self right now and could use some kind of support to move away from this toxic behavior," she suggests.

Measures

Negative Moral Judgments:
Participants rated their agreement (1—7; 1 = "Strongly disagree", 7 = "Strongly agree") with the following statements:

- I believe X was Antisemitic.
- I believe X intended to be Antisemitic.
- I blame X for being Antisemitic.

Desire to Punish:
Participants rated their agreement (1—7; 1 = "Strongly disagree", 7 = "Strongly agree") with the following statements:

- I would like to punish X.
- I have the impulse to attack X.
- I have the urge to insult X.
- I have the urge to say something nasty to X.

DV

Participants read the following information about a retributive donation campaign:

> The Anti-Defamation League, a prominent Jewish organization, is currently soliciting donations to fight Kanye's antisemitism.
>
> **All donations will go towards pressuring companies with sponsorship relationships with Kanye to cut ties, and financially punish Kanye for his behavior.**
>
> How likely are you to donate to this cause?

Participants then rated their likelihood of donation on a 1—7 scale (1 = “extremely unlikely”, 7 = “extremely likely”)

Study 5

Stimuli

Volitional Wrongdoing Present vs. Absent conditions: same as Study 3

Measures

Negative Moral Judgments: Same as Study 4 with context updated.

Negative Emotions: We used a scale measure of Contempt, Anger, and Disgust (CAD) adapted from (Karppinen, King, and Russell 2023). Participants rated the extent to which the stimuli they were presented with made them feel the following emotions:

- Contempt emotions
    - Contempt
    - Disdain
    - Scorn
- Anger
    - Angry
    - Frustrated
    - Furious
- Disgust Emotions
    - Disgusted
    - Repulsed
    - Sickened

DV

Same as Study 3 (Retributive Option Present condition)

Study 6

Stimuli

Left-wing story: Same as Study 3

Right-wing story: Same as Post-Test Study 1

Measures

Left-Wing Authoritarianism: We used the following Left Wing Authoritarianism scale, adapted from Costello and Patrick (2021) and Costello et al. (2022) to measure authoritarianism. This scale uses a series of 7-point Likert scale items (1 = "Strongly disagree"; 7 = "Strongly agree"):

- The rich should be stripped of their belongings and status.
- We would be much better off if all of the rich people were at the bottom of the social ladder.
- When the tables are turned on the oppressors at the top of society, I will enjoy watching them suffer the violence that they have inflicted on so many others.
- We need to replace the established order by any means necessary.
- Anyone who opposes gay marriage must be homophobic.
- People who are truly worried about terrorism should shift their focus to the nutjobs on the far-right.
- The "old-fashioned ways" and "old-fashioned values" need to be abolished.
- All political conservatives are fools.
- Classroom discussions should be safe places that protect students from disturbing ideas.
- University authorities are right to ban hateful speech from campus.
- To succeed, a workplace must ensure that its employees feel safe from criticism.
- I am in favor of allowing the government to shut down right-wing internet sites and blogs that promote nutty, hateful positions.
- Getting rid of inequality is more important than protecting the so-called "right" to free speech.

Right-Wing Authoritarianism: We used the Right-Wing Authoritarianism scale as described by Altemeyer (1996). The scale is comprised of a series of 7-point Likert scale items (1 = "Strongly disagree"; 7 = "Strongly agree"; note that many items are reverse-coded):

- The established authorities generally turn out to be right about things, while the radicals and protestors are usually just "loud mouths" showing off their ignorance.
- Women should have to promise to obey their husbands when they get married.
- Our country desperately needs a mighty leader who will do what has to be done to destroy the radical new ways and sinfulness that are ruining us.
- Gays and lesbians are just as healthy and moral as anybody else.
- It is always better to trust the judgment of the proper authorities in government and religion than to listen to the noisy rabble-rousers in our society who are trying to create doubt in people's minds.
- Atheists and others who have rebelled against the established religions are no doubt every bit as good and virtuous as those who attend church regularly.
- The only way our country can get through the crisis ahead is to get back to our traditional values, put some tough leaders in power, and silence the troublemakers spreading bad ideas.
- There is absolutely nothing wrong with nudist camps.
- Our country needs free thinkers who have the courage to defy traditional ways, even if this upsets many people.
- Our country will be destroyed someday if we do not smash the perversions eating away at our moral fiber and traditional beliefs.
- Everyone should have their own lifestyle, religious beliefs, and sexual preferences, even if it makes them different from everyone else.
- The "old-fashioned ways" and the "old-fashioned values" still show the best way to live.
- You have to admire those who challenged the law and the majority's view by protesting for women's abortion rights, for animal rights, or to abolish school prayer.
- What our country really needs is a strong, determined leader who will crush evil, and take us back to our true path.
- Some of the best people in our country are those who are challenging our government, criticizing religion, and ignoring the "normal way things are supposed to be done."
- God's laws about abortion, pornography and marriage must be strictly followed before it is too late, and those who break them must be strongly punished.

- There are many radical, immoral people in our country today, who are trying to ruin it for their own godless purposes, whom the authorities should put out of action.
- A "woman's place" should be wherever she wants to be. The days when women are submissive to their husbands and social conventions belong strictly in the past.
- Our country will be great if we honor the ways of our forefathers, do what the authorities tell us to do, and get rid of the "rotten apples" who are ruining everything.
- There is no "one right way" to live life; everybody has to create their own way.
- Homosexuals and feminists should be praised for being brave enough to defy "traditional family values."
- This country would work a lot better if certain groups of troublemakers would just shut up and accept their group's traditional place in society.

DV

Left-wing DV: Same as Study 3

Right-wing DV: Participants read the following statement:

> An organization is currently raising funds to support the students and parents who feel harmed by John Gerber's speech:
>
> The **Kentucky Concerned Parents Alliance** promises to use all funds raised to support family-friendly education at Kentucky middle schools. **For every donation, the organization also promises to send a letter to the middle school in question calling for John Gerber's dismissal.**
>
> Would you be willing to donate to this organization?

Participants then rated their willingness to donate to the organization on a 1—7. Scale (1 = “Definitely not”, 7 = “Definitely yes”)

Study 7

Stimuli

Volitional Wrongdoing Present vs. Absent conditions: same as Study 3.

High Efficacy Condition

Prior to being presented with the DV, Participants in this condition read the following text:

> "In response to this incident, the Kentucky Antiracist Students Alliance (KASA) is raising funds to support those harmed by the professor's speech. **For each donation, KASA promises to send a letter calling for the professor's dismissal.**
>
> The University has responded to these efforts:
>
> "The volume of letters we have received is making us seriously reconsider Professor Gerber's future employment at our university.""

Low Efficacy Condition

Prior to being presented with the DV, Participants in this condition read the following text:

> "In response to this incident, the Kentucky Antiracist Students Alliance (KASA) is raising funds to support those harmed by the professor's speech. **For each donation, KASA promises to send a letter calling for the professor's dismissal.**
>
> The University has responded to KASA's efforts:
>
> "We do not make employment decisions on the basis of letters from the public.""

Measures

Negative Moral Judgments: Same as Study 4.

DV

Participants were asked to "rate your likelihood of donating to KASA below", on a 1—7 scale (1 = "extremely unlikely", 7 = "extremely likely")

Post-Test 1 (Method & Results in Web Appendix D)

Stimuli

Volitional Wrongdoing Present vs. Absent manipulation: Same as Study 3

Measures

DV

"I believe Professor Gerber deliberately chose to say the N-word" (1—7; 1 = "Strongly disagree", 7 = "Strongly agree")

Post-Test 2 (Method & Results in Web Appendix E)

Stimuli

Volitional Wrongdoing Present vs. Absent manipulation: Same as Study 4

Measures

DV

"I believe Kanye was in control of his actions when he made antisemitic statements" (1—7; 1 = "Strongly disagree", 7 = "Strongly agree")

<u>Supplementary Study 1 (Method & Results in Web Appendix F)</u>

Stimuli

Volitional Wrongdoing Present vs. Absent manipulation: Same as Study 4

Measures

Negative Moral Judgments: Same as Study 4

Desire to Punish: Same as Study 4

DV

Same as Study 4

Post-Test Study 1 (Method & Results in Web Appendix H)

Stimuli

Ukraine War: Same as Supplemental Study 5

Professor saying the N-word: Same as Study 3

Professor discussing transgender identity:

A teacher at a Kentucky middle school is currently being protested by several parents rights groups for pushing transgender ideology on students.

Late last month, John Gerber, the teacher, was teaching his students in a sexual education class, which typically provides students with information about practicing safe sex and abstinence.

"Some little girls and boys feel as though they are born in the wrong body and want to change genders," Gerber said, according to a recording of one of the students. **Gerber then told the middle schoolers that if any of them were questioning their gender identity and considering gender transition, they should come to him if their parents were unsupportive**.

Gerber, who has worked with transgender youth but is not himself transgender, did not warn students or parents that he would be discussing gender ideology in the classroom. And some students and parents were concerned about what they heard Gerber discuss in class. So they wrote a letter to the principal of the school, Kyle Karminsky, describing Gerber as a danger to students for threatening to encourage their gender transitions without parental consent.

"What we heard in class was an existential threat to our children," wrote the parents, who identified themselves as Concerned Parents for the Protection of our Children. "There are many different ways to introduce children to transgender ideology, but John Gerber's decision to discuss this without consent of us parents is unacceptable to our middle school community. The negligence and disregard displayed by John Gerber was very clear in today's class."

The students said some of them had voiced their concern to John Gerber during class, but that he'd **deliberately** made similar statements in following classes anyway. They also said he **justified** his pro-trans statements by claiming that "our mission as teachers is to support our students, whatever their gender is", and that he "hoped some parents are made uncomfortable by the class, because parents scared of their children learning about being transgender are themselves in need of education."

Measures

Identity Threat: Participants completed a measure of identity threat, adapted from Breakwell and Jaspal (2022). Participants were asked to think about the article they just read and to rate their agreement with a series of 7-point Likert scales (1 = "Strongly disagree"; 7 = "Strongly agree"):

- It undermines my sense of self-worth.
- It makes me feel less competent.
- I feel that my identity has changed.
- It makes me feel less unique as a person.

Political Valence: For the Professor saying the N-word and Professor discussing transgender identity stimuli, participants were asked to rate "Which group would you expect to be most upset by the professor saying the N word?":

- Left-wing voters
- Right-wing voters
- Neither/Both

Supplementary Study 2 (Method & Results in Web Appendix I)

Stimuli

Same as Study 3

Measures

Left-Wing Authoritarianism: Same as Study 6

Status-Seeking

We used the following Moral Grandstanding Motivations scale to measure status-seeking, adapted from Grubbs et al. (2019), is comprised of a series of 7-point Likert scale items (1 = "Strongly disagree"; 7 = "Strongly agree"). This scale is split into two sub-dimensions: Prestige-strivings and Dominance-strivings.

Prestige-Strivings:

- I hope that my beliefs cause other people to want to share those beliefs.
- I am particularly good at sharing my beliefs.
- My beliefs should be inspiring to others.
- I often share my beliefs in the hope of inspiring people to be more passionate about their beliefs.
- I want to be on the right side of history about moral/political issues.
- Even if expressing my views does not help anyone, it is important that I share them.

Dominance-Strivings:

- When I share my beliefs, I do so to show people who disagree with me that I am better than them.
- I share my beliefs to make people who disagree with me feel bad.
- When I share my beliefs, I do so to shame people who do not share those beliefs.
- When I share my beliefs, I do so in the hope that people different than me will feel ashamed of their beliefs.

DV

Same as Study 3, Retributive Option Present condition.

Supplementary Study 3 (Method & Results in Web Appendix J)

Stimuli

Volitional Wrongdoing Present vs. Absent conditions: same as Study 3

Measures

Negative Moral Judgments: Same as Study 4 with context updated.

Left-Wing Authoritarianism: Same as Study 6

Moral Identity: We used the following moral identity scale, developed by Aquino and Reed (2003). Participants first read the following prompt:

> *Listed below are some characteristics that may describe a person: Caring, Compassionate, Fair, Friendly, Generous, Hardworking, Helpful, Honest, Kind*
>
> *The person with these characteristics could be you or it could be someone else. For a moment, visualize in your mind the kind of person who has these characteristics. Imagine how that person would think, feel, and act. When you have a clear image of what this person would be like, answer the following questions.*

The scale then uses a series of 7-point Likert scale items (1 = “Strongly disagree”; 7 = “Strongly agree”):

- It would make me feel good to be a person who has these characteristics.
- Being someone who has these characteristics is an important part of who I am.
- A big part of my emotional well-being is tied up in having these characteristics.
- I would be ashamed to be a person who has these characteristics.
- Having these characteristics is not really important to me.
- Having these characteristics is an important part of my sense of self.
- I strongly desire to have these characteristics.
- I often buy products that communicate the fact that I have these characteristics.
- I often wear clothes that identify me as having these characteristics.
- The types of things I do in my spare time (e.g., hobbies) clearly identify me as having these characteristics.
- The kinds of books and magazines that I read identify me as having these characteristics.

- The fact that I have these characteristics is communicated to others by my membership in certain organizations.
- I am actively involved in activities that communicate to others that I have these characteristics.

Reactance: We used the following scale from Hong and Page (1989) to measure participants' reactant personalities. The scale uses a series of 7-point Likert scale items (1 = "Strongly disagree"; 5 = "Strongly agree"):

- Regulations trigger a sense of resistance in me.
- I find contradicting others stimulating.
- When something is prohibited, I usually think "that's exactly what I am going to do."
- The thought of being dependent on others aggravates me.
- I consider advice from others to be an intrusion.
- I become frustrated when I am unable to make free and independent decisions.
- It irritates me when someone points out things which are obvious to me.
- I become angry when my freedom of choice is restricted.
- Advice and recommendations usually induce me to do just the opposite.
- I am contended only when I am acting of my own free will.
- I resist the attempts of others to influence me.
- It makes me angry when another person is held up as a role model for me to follow.
- It disappoints me to see others submitting to society's standards and rules.

DV

Participant then read the following statement:

> Two organizations are raising funds in response to the controversy over Professor Gerber's class:
>
> **The Western Kentucky Black Students Group** promises to use all funds raised to run a series of anti-racist workshops for students and faculty.
>
> **The Kentucky Antiracist Students Alliance** promises to use all funds raised to run a series of anti-racist workshops for students and faculty. **For every donation, the organization also promises to send a letter to the University calling for Professor Gerber to be fired to punish him for his racist behaviour.**

Participants were asked to "Please rate your preference of organization to donate to", on a 1—6 scale, with WKSB (1) and KASA (6) as scale anchors.

Supplementary Study 4 (Method & Results in Web Appendix K)

Stimuli

Volitional Wrongdoing Present vs. Absent conditions: same as Study 4

Measures

Reactance: Same as Supplementary Study 3

Moral Identity: Same as Supplementary Study 3

DV

Same as Study 4

Supplementary Study 5 (Method & Results in Web Appendix L)

Stimuli

Initial Story:

Air raid sirens are sounding out across multiple regions in Ukraine again on Tuesday with the emergency services warning of more Russian strikes, a day after a series of Russian attacks left at least 19 people dead and over 100 injured.

Ukrainian officials reported that energy infrastructure in the western city of Lviv had been hit earlier, while the city of Zaporizhzhia in the south was also targeted this morning.

Ukrainian President Volodymyr Zelenskyy said in his nightly address Monday that Ukraine will not be intimidated by the strikes that took place Monday and which targeted various regions including the capital Kyiv. Urgent work was being done to repair and restore power supplies damaged during the strikes, he added.

The multiple attacks by Russia came several days after a blast partially destroyed the Kerch Bridge that links the Russian mainland to Crimea, which Moscow illegally annexed in 2014.

Kyiv has not said whether it was responsible for the attack on the bridge, although the blast was widely seen as humiliating for Moscow and President Vladimir Putin. The leaders of the Group of Seven of the world's most developed economies held an emergency meeting Tuesday to discuss Russia's war in Ukraine. Addressing the meeting via videolink, Zelenskyy asked for more air defense weapons.

Top officials in the United States, European Union and at the United Nations expressed shock and horror Monday over the strikes. U.N. Secretary-General Antonio Guterres was "shocked" by the attacks, saying through a spokesperson that they represented an escalation of the war.

The strikes have damaged significant parts of Ukraine's energy grid, prompting the nation's energy ministry to announce it would halt exports of electricity to the EU starting Tuesday.

Retributive Donation Option

A recent initiative by Ukrainian charities have caught mainstream media attention. These charities are offering the opportunity for people across the world to leave custom messages on artillery shells in possession of the Ukrainian military.

In exchange for a donation of $10 to any participating Ukrainian charity, Ukrainian soldiers will write a custom message on artillery shells that will be shot at real Russian targets. Many individuals have taken this opportunity to leave messages expressing anger at the Russian military (see below).

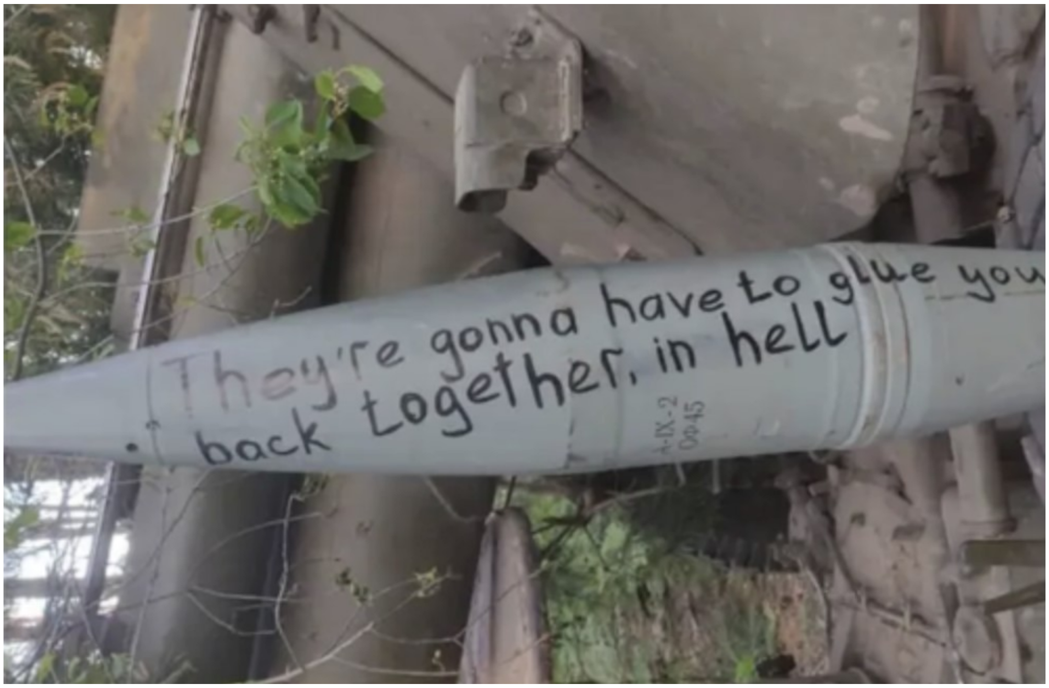

This initiative has proved beneficial for Ukrainian charities, resulting in over $500,000 in donations for Ukraine. This money has resulted in many more Ukrainians having access to necessary medical care, food, and shelter.

For-Profit Payment Option

A recent initiative by a company has caught mainstream media attention. This company is offering the opportunity for people across the world to leave custom messages on artillery shells being sent to the Ukrainian military.

In exchange for a payment of $10, The company will write a custom message on artillery shells that will be shot at real Russian targets. Many individuals have taken this opportunity to leave messages expressing anger at the Russian military (see below).

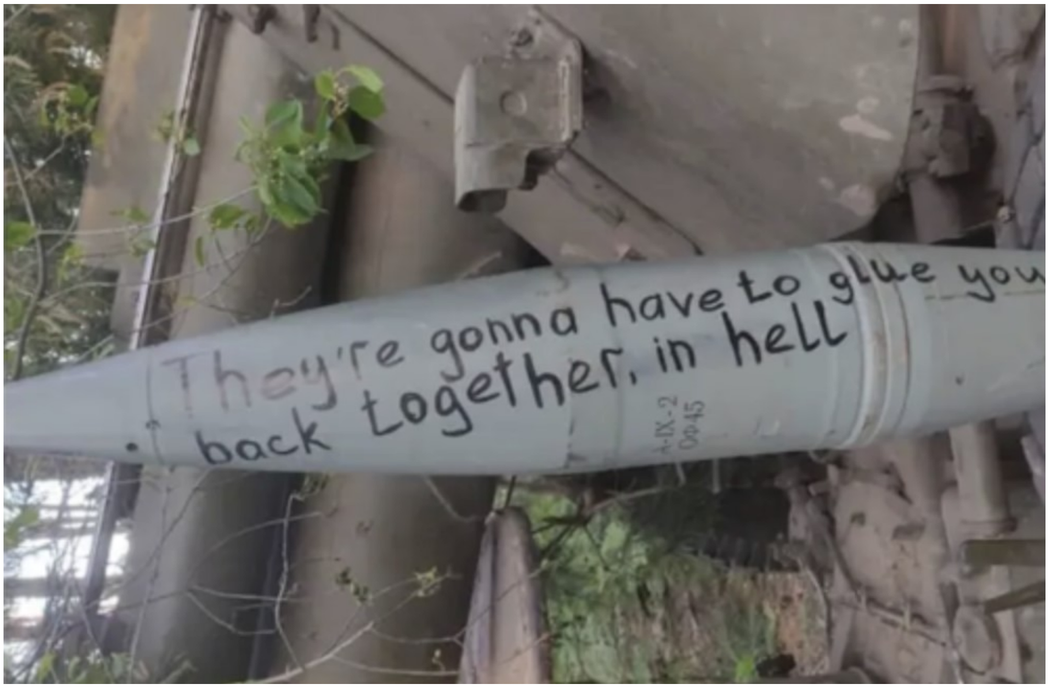

This initiative has proved profitable for the company, resulting in net cash flow of over $500,000. This has led to significant increases in the company's bottom line, contributing to their 13% Year-over-Year growth in fourth-quarter operating income.

Measures

Perceived Risk:

Participants rated their agreement (1—7; 1 = "Strongly disagree", 7 = "Strongly agree") with the following statements:

- Purchasing this service would be socially risky.
- Purchasing this service would be legally risky.
- Purchasing this service would be professionally risky.

DV

Depending on experimental condition, participants were asked to respond to one of the following 7-point scales (1 = "Extremely unlikely", 7 = "Extremely likely").

For-Profit Payment Option

"How likely would you be to pay $10 in exchange for a custom message on an artillery shell?"

Retributive Donation Option

"How likely would you be to donate $10 in exchange for a custom message on an artillery shell?"

Web Appendix D: Post-Test Study 1

The purpose of this study is to post-test the stimuli used in Studies 3, 5, and 7. Specifically, this study tests the extent to which our volitional wrongdoing manipulations affects participants' perception that the wrongdoer in question was in control of their actions and acting in a volitional manner.

Method

Prolific Academic participants (N = 200, M_{Age} = 39.60, 50.00 % female) completed this study in exchange for financial compensation. Participants were exposed to the same experimental manipulation used in Study 3: a professor either 1) saying the N-word to students, or 2) saying a Chinese word that sounds like the N-word to students. Participants then rated their agreement with a manipulation check that read: "I believe Professor Gerber deliberately chose to say the N-word" (1—7; 1 = "Strongly disagree", 7 = "Strongly agree").

Results and Discussion

Consistent with our theorizing, participants in the volitional wrongdoing condition indicated higher agreement with our manipulation check (M = 6.05) than those in the non-volitional wrongdoing condition (M = 2.50; difference = 3.55, $t(178.02) = 16.73$, $p < .001$).

These results indicate that our manipulation of volitional wrongdoing in Studies 3, 5, and 7 did indeed affect participants' perceptions of volitional wrongdoing.

Web Appendix E: Post-Test Study 2

The purpose of this study is to post-test the stimuli used in Study 4. Specifically, this study tests the extent to which our volitional wrongdoing manipulations affects participants' perception that the wrongdoer in question was in control of their actions and acting in a volitional manner.

Method

Prolific Academic participants (N = 200, M_{Age} = 39.79, 50.00 % female) completed this study in exchange for financial compensation. Participants were exposed to the same experimental manipulation used in Study 4: and article where Kanye West's antisemitic statements were characterized as either related or unrelated to mental illness. Participants then rated their agreement with a manipulation check that read: "I believe Kanye was in control of his actions when he made antisemitic statements" (1—7; 1 = "Strongly disagree", 7 = "Strongly agree").

Results and Discussion

Consistent with our theorizing, participants in the volitional wrongdoing condition indicated higher agreement with our manipulation check (M = 5.43) than those in the non-volitional wrongdoing condition (M = 4.34; difference = 1.09, t(182.66) = 5.38, $p < .001$).

These results indicate that our manipulation of volitional wrongdoing in Study 4 did indeed affect participants' perceptions of volitional wrongdoing.

Web Appendix F: Supplementary Study 1 (Study 4 Follow-up)

The primary goal of this supplementary study was to directly replicate the serial mediation pathway observed in Study 4, using an alternative sample pool. Whereas in Study 4 we specifically recruited Jewish and non-Jewish participants to test identity threat as an alternative explanation of our effects, in this study we test a similar serial mediation model using an ordinary Prolific sample.

Method

Prolific Academic participants ($N = 1199$, ages 18-93, $M_{age} = 41.27$, 49.96% female) completed this study in exchange for financial payment and were randomly assigned to conditions in a 2-factor (wrongdoing: volitional vs. non-volitional) between-participants design. Our data collection and analysis plan was pre-registered at https://aspredicted.org/gjsx-yypg.pdf. One participant was excluded for not completing all measures, consistent with our pre-registration. The results below do not materially change with or without their inclusion.

Participants were exposed to the same stimuli and manipulation used in Study 4, and answered the same measures of negative moral judgments, desire to punish, and likelihood of making a retributive donation. Our full list of measures can be found in Web Appendix C.

Results and Discussion

We first observed a direct main effect of our experimental condition, such that participants were more willing to retributively donate when wrongdoing was volitional

($M_{Likelihood}$ = 3.21) relative to when wrongdoing was non-volitional ($M_{Likelihood}$ = 2.93; t(1195.3) = 2.54, p = .011).

We next used Hayes (2013)'s PROCESS Model 6, with bootstrap re-sampling (*N*=10,000; Figure W1) to test the effect of perceived volitional wrongdoing by Kanye West on donation likelihood through negative moral judgment and desire to punish. First, as expected, we found a significant effect of our manipulation on participants' negative moral judgments of West (β = .256, SE = 0.042, CI_{95}: .174, .339, t(1197) = 6.10, p < .001). Specifically, participants in the volitional wrongdoing condition (M_{NMJ} =5.59) had higher negative moral judgments of West than those in the non-volitional wrongdoing condition ($M_{NMJ =}$ 5.08). Next, controlling for experimental condition, negative moral judgments significantly and positively predicted participants' desires to punish West (β = .377, SE = .026, CI_{95}: .327, .428, t(1196) = 14.68, p < .001). Finally, negative moral judgments (β = .212, SE = 0.039, CI_{95}: .137, .288, t(1195) = 5.52, p < .001) and desire to punish (β = .452, SE = 0.040, CI_{95}: .374, .530, t(1195) = 11.36, p < .001), in turn, each increased participants' willingness to make retributive donations.

The total indirect effect on likelihood to make a retributive donation was significant (β = .111, SE = 0.025, CI_{95}: .063, .160). In line with our conceptualization, we found an indirect effect of volitional wrongdoing on donation likelihood through negative moral judgments and desire to punish (β = .044, SE = 0.009, CI_{95}: .028, .061).

Figure W1: Supplementary Study 1 Results

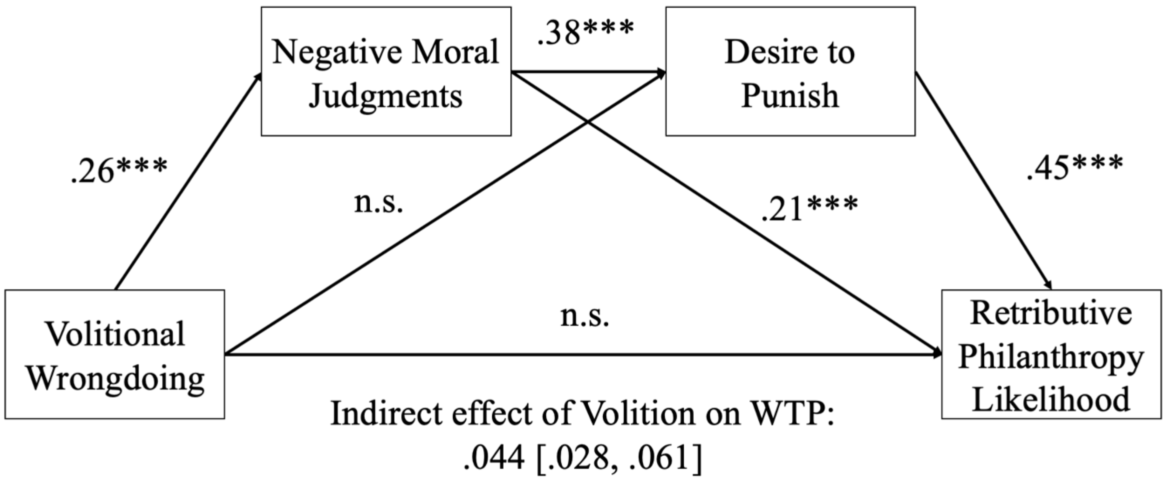

Web Appendix G: Study 5 Additional Analysis

In Study 5, we report the results of several serial mediation models which position other-condemning moral emotions as mediating the effect of negative moral judgments on retributive philanthropy likelihood. However, such analyses presume that moral judgments of wrongdoing precede emotional reactions to wrongdoing, whereas some intuitionist accounts of moral judgments suggest that emotions precede judgments (e.g., Haidt, 2001). We therefore report the results of several pre-registered (https://aspredicted.org/t5kr-j9q7.pdf) follow-up models, which reverse the serial mediation order between negative moral judgments and other-condemning moral emotions reported in Study 5.

Specifically, we tested whether participants' negative moral judgments mediated the effect of other-condemning moral emotions (i.e., contempt, anger, and disgust) on retributive donation using Hayes PROCESS Model 6 with bootstrap resampling (N=10,000). We found that other-condemning moral emotions positively predicted increased negative moral judgments (β = .555, SE = .023, CI_{95}: .511, .600, t(1196) = 24.60, $p < .001$), which in turn predicted higher likelihood of making a retributive donation (β = .467, SE = .030, CI_{95}: .409, .527, t(1195) = 15.64, $p < .001$). We observed an overall significant index of serial mediation that excluded zero (β = .238, SE = .031, CI_{95}: .180, .301), suggesting that negative moral judgments significantly mediate the relationship between other-condemning moral emotions and donation. We visualize these results in Figure W2.

Figure W2: Study 5 Results

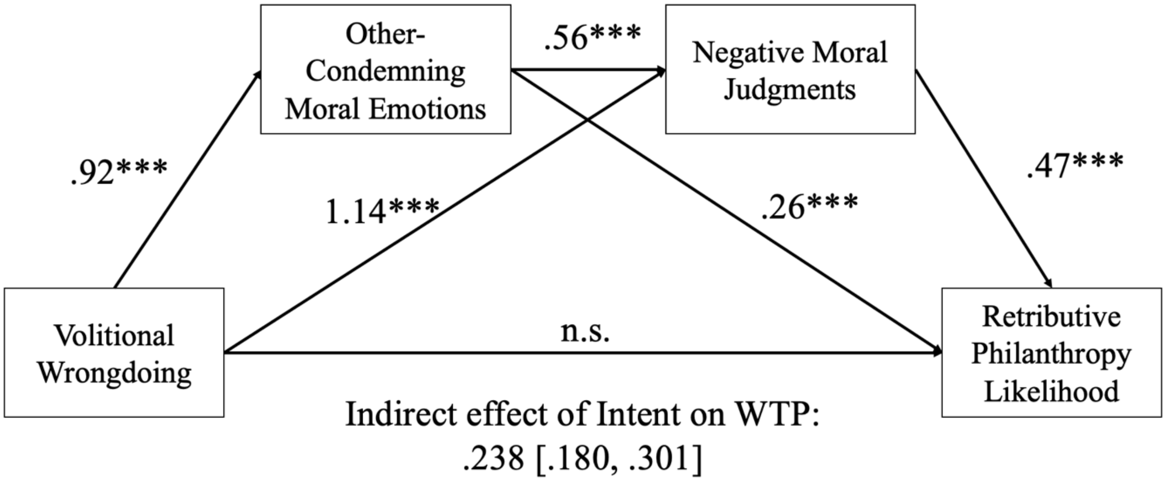

In several complementary pre-registered PROCESS Model 6 analyses, we estimate the mediating effect of each sub-component of our other-condemning moral emotions scale (contempt, anger, disgust) and find consistent results across each, such that contempt (β = .240, SE = .032, CI_{95}: .181, .306), anger (β = .177, SE = .028, CI_{95}: .126, .234), and disgust (β = .258, SE = .032, CI_{95}: .198, .324) all significantly mediated the effect of intent on retributive donation, and the effects of these emotions were in turn mediated by negative moral judgments.

Web Appendix H: Post-Test Study 3

The purpose of this study is to post-test the stimuli used in our studies. Specifically, this study tests whether our stimuli threaten participants' identity, as well as tests the political valence of some of our stimuli. We expected that participants would generally disagree that the stimuli we use is particularly threatening to their identity, and that the issues we selected for our studies will be accurately classified as left-wing or right-wing norm violations.

Method

Undergraduate participants (N = 398, M_{Age} = 19.01, 75.76% female), drawn from the same pool as Study 3 completed this study in exchange for course credit. Participants all read three different stimuli used throughout our manuscript and web appendix: the Ukraine war story (Supplemental Study 5), the story of a professor using the N-word (Studies 3, 5, 6, 7), and the story of a professor exposing students to transgender ideology (Study 6). For each story, participants completed a 4-item, 7-point scale (1 = "Strongly disagree", 7 = "Strongly agree"; e.g., "it undermines my sense of self-worth") measure of identity threat. For the latter two stories, which are used in Study 6 to test the effects of ideological congruency and authoritarianism, we also asked participants to rate which group would be most upset by the story: Left-wing voters, Right-wing voters, or both/neither.

Results and Discussion

Consistent with our theorizing, participants generally did not consider our stimuli threatening to their identities. Participants' average identity threat responses were

significantly below the scale midpoint of four for the Ukraine war story ($M_{Threat} = 2.78$, $t(394) = -19.87$, $p < .001$), the story of the professor using the N-word ($M_{Threat} = 2.80$, $t(394) = -18.50$, $p < .001$), and the story of the professor exposing students to transgender ideology ($M_{Threat} = 2.72$, $t(394) = -20.47$, $p < .001$). Additionally, participants recognized that the story of a professor using the N-word mostly violated left-wing norms (57.07% left, 31.06% both/neither, 11.36% right) and that the story of a professor exposing students to transgender ideology mostly violated right-wing norms (14.90% left, 21.21% both/neither, 63.38% right).

These results provide support for our theorizing that our observed and hypothesized effects in our main manuscript are unlikely to be driven by identity threat responses, because participants did not find our stimuli threatening to their identity. Additionally, these results give us confidence that our left-wing and right-wing norm violations in Study 6 were appropriately selected.

Web Appendix I: Supplementary Study 2 (Study 6 Authoritarianism Follow-Up Study)

In this follow-up study, we build on the results of Study 6 by replicating the moderating effect of authoritarianism in an incentive-compatible design, wherein participants choose between making a retributive donation, a normal donation, or not donating at all. We also rule out status-seeking as an alternative explanation. Status-seeking is a plausible alternative explanation, because there are clear social rewards for aggressive behavior (Tosi and Warmke 2016), and individuals learn to behave aggressively in order to seek status (Brady et al. 2021).

Method

Undergraduate participants (N = 375, M_{age} = 19.12, 51.46% female, 48.00% male, 0.54% nonbinary) from a large North American university completed this study for course credit. Seven participants failed to complete this study in its entirety and were thus excluded. The study followed a 2 (wrongdoing: volitional vs. non-volitional) x continuous (authoritarianism) between-participants design. Participants first completed a set of scales that measure authoritarian and status-seeking traits. Authoritarianism was measured using Costello et al.’s (2022) “Left-Wing Authoritarianism” scale, which is appropriate given that our sample is comprised of generally liberal college students, and our stimuli concern violations of traditionally left-wing norms against racism (see Web Appendix H for more details). Status-seeking was measured using Grubbs et al.'s (2019) “Moral Grandstanding Motivations” (MGM) scale (see Web Appendix C for additional information on these measures).

Next, to separate the focal measures from the primary task, participants completed a neutral picture-rating filler task for approximately five minutes. Finally, participants were presented with the same stimuli used in Study 3: an article of a professor who either said a racial slur or another word that sounded like a racial slur. As in Study 3, participants were given a $1 cash bonus that they could either 1) keep, 2) donate to a retributive charity, or 3) donate to a non-retributive charity. In this study, a retributive option was present for all participants. We predicted participants would be most likely to make retributive donations when wrongdoing was described as volitional, and this effect would be strongest for participants higher in authoritarianism

Results

We first compared participants' choices to keep their bonus, make a traditional donation, or make a retributive donation between conditions. Compared to the non-volitional condition, the proportion of retributive donors rose in the volitional condition (difference = 10.8%, $\chi^2 = 5.76$, $p = .016$), while the proportion of those keeping their bonuses fell (difference = 10.0%, $\chi^2 = 4.94$, $p = .026$). We observed no effect of experimental condition on the proportion of participants choosing to make a traditional donation (difference = 0.6%, $\chi^2 < 0.001$, $p = .99$). Thus, perceiving a wrongdoing as volitional led to more overall donations (H1a), and this increase was driven by participants making retributive donations, with no observable cannibalization effects on normal donations. These results suggest that perceiving a wrongdoing as volitional coupled with an option for retribution can capture donors who would not have otherwise donated. Put another way, retributive options can

increase overall donations in the presence of a volitional wrongdoer, an important finding for charitable marketers.

We then conducted a multinomial logistic regression, estimating the likelihood of participants choosing between three possible options: 1) keeping their bonus, 2) making a normal donation, and 3) making a retributive donation. Consistent with Study 3, we analyzed our data using "normal donation" as our base choice, with coefficients representing the effect of volitional wrongdoing, authoritarianism, and status-seeking motives on participants' preference for keeping their bonus or making retributive donations relative to making a normal donation. We use normal donation as our base choice, as it was the most popular option, and because it allows us to directly compare consumer preference for retributive versus normal donations. All estimates are shown in Table W2.

Table W2: Multinomial Logistic Regression of Donor Choice

Parameter	Coefficient	SE	95% CI	z	p-value
**Retributive**					
(Intercept)	-0.38	1.10	-2.53, 1.77	-0.35	0.729
Volition	-2.58	1.31	-5.16, -0.01	-1.97	0.049
Authoritarianism	-0.33	0.27	-0.97, 0.10	-1.61	0.107
Status-seeking	0.21	0.19	-0.17, 0.58	1.07	0.284
Intent x Authoritarianism	0.84	0.35	0.15, 1.52	2.39	0.017
**Keep**					
(Intercept)	0.20	0.98	-1.73, 2.13	0.20	0.839
Volition	-1.15	1.17	-3.45, 1.15	-0.98	0.327
Authoritarianism	-0.89	0.24	-1.35, -0.42	-3.75	<0.001
Status-seeking	0.59	0.20	0.21, 0.98	3.04	0.002
Intent x Authoritarianism	0.16	0.34	-0.50, 0.82	0.46	0.463

Central to our theorizing, we found a significant interaction, such that when a wrongdoing is volitional, authoritarianism positively predicts retributive donation choice (β = 0.84, SE = 0.35, CI_{95}: 0.15, 1.52, p = .017). This finding complements results from Study 6 and also supports our prediction that authoritarian personalities seem to be more willing to make retributive donations in cases where there is volitional wrongdoing. Importantly, we found no evidence for the effect of status-seeking motivation on donation choice, which suggests that retributive donations are not pursued for status.

Discussion

These results conceptually replicate and are consistent with the findings of Study 6. Namely, that authoritarianism explains, in part, why individuals elect to make retributive donations. Authoritarianism as an influential factor is consistent with a retribution account of our observed phenomenon, as authoritarianism is traditionally associated with punitive behaviors.

Additionally, these results suggest that the effects of authoritarianism on donation manifest above and beyond the effect of status-seeking. This serves to distinguish retributive philanthropy from modern cancel culture, which is typically understood as involving status-seeking individuals seeking to punish others with little to no regard for whether they meant to do wrong (Lukianoff and Haidt, 2018; Lukianoff and Schlott, 2023; Tosi and Warmke, 2016).

Web Appendix J: Supplementary Study 3 (Personality Follow-up Study)

In this study, we provide additional process support for our retribution account via individual-difference moderation. Specifically, we replicate the findings of Study 6 which show that authoritarianism can be positively related to retributive donations. Additionally, we sought to rule out moral identity and reactance as potential alternative explanations for our observed effect of volitional wrongdoing on retributive donation. Our reasoning for exploring these two alternative explanations is described below.

We conceptualize retributive philanthropy as an emerging phenomenon and contend that our retribution account for donating is theoretically distinct from prior prosocial motives, because it is driven by negative moral judgments of volitional wrongdoers and a desire to punish them. However, we acknowledge that our predictions could be perceived as consistent with alternative prosocial frameworks. For example, it is possible that consumers high in moral identity (Aquino and Reed 2003) may be more likely to make retributive donations given that such donations involve moral judgments, and prior work has shown how moral identity is positively related to donation behavior (Winterich et al. 2013). While we agree consumers' moral identities are relevant to their donation decision-making, we do not believe that this account can explain our empirical findings. Our retribution account predicts that consumers will engage in retributive donations when they perceive wrongdoing is volitional, and that this increase in donation will result even among consumers regardless of their moral identity.

Moreover, because many of the real-world wrongdoings highlighted in the introduction of the main manuscript, and many wrongdoings examined in our empirical section concern threats to individual rights and freedoms, it is plausible that feelings of reactance (Brehm 1966) may account for the donation behavior we observe. Consumers may perceive retributive donations as a means to restore or regain a sense of control. We acknowledge that a desire to restore freedom can and does lead to hostile reactions to restore freedom and may be conceived as plausible in our donation context; however, we contend reactance cannot explain retributive donations over and above our retribution account. While some real-world retributive philanthropy campaigns—and a small portion of those used in our studies—involve a threat to freedom, others do not. Interestingly, of those situations that threaten freedom, many (if not most) involve threats to the freedoms of other people and not the retributive donors. Reactance is typically understood to concern threats to one's *personal* freedoms, rather than the vicarious experiences of others' freedoms being threatened (Miron and Brehm 2006). For these reasons, we contend a theoretical framework centered on reactance cannot better explain our results.

We explore each of authoritarianism, reactance, and moral identity in turn in this study. Specifically, we test whether each of these individual differences interacts with our theoretical framework, such that individuals high in each trait are more sensitive to volitional wrongdoing and therefore form stronger negative moral judgments of wrongdoers.

Method

Prolific Academic participants (N = 1176, ages 18-85, M_{age} = 44.66, 50.25% female) completed this study in exchange for financial payment and were randomly assigned to conditions in a 2-factor (wrongdoing: volitional vs. non-volitional) between-participants design. Our data collection and analysis plan were pre-registered at https://aspredicted.org/994v-tcfw.pdf. Per our pre-registration, nine participants failed to complete the study in its entirety and were excluded, though our results do not materially change with their inclusion.

Participants first completed three personality measures in randomized order: Costello et al.'s (2022) Left Wing Authoritarianism scale (LWA), Hong and Page's (1989) Psychological Reactance scale, and Aquino and Reed's (2003) Moral Identity scale. Participants were then exposed to the same stimuli used in Study 3 and completed the same measure of negative moral judgments used in Study 4 (See Web Appendix C for information on all scales and measures).

Finally, participants were asked to choose between donating to one of two organizations (same as Study 3) on a six-point scale, with each organization serving as a scale anchor: the Western Kentucky Black Students Group (1), and the Kentucky Antiracist Students Alliance (6). Both organizations were described as offering similar charitable benefits. The Kentucky Antiracist Students Alliance was additionally described as promising to send a letter to the dean calling for the professor's firing for every donation received.

Results

We first replicated our established mediation process (PROCESS Model 4, 10,000 resamples). Our experimental manipulation had a positive effect on negative moral judgments (β = 1.358, SE = .102, CI_{95}: 1.159, 1.557, t(1174) = 13.39, p < .001), which in turn increased subsequent willingness to make a retributive donation (β = .365, SE = .026, CI_{95}: .314, .416, t(1173) = 14.07, p < .001). The effect of volitional wrongdoing on retributive donation was fully mediated by negative moral judgments (β = .495, SE = .053, CI_{95}: .397, .607).

We next tested the moderating effects of each of the three personality measures on the effect of volitional wrongdoing on negative moral judgments (PROCESS model 7, 10,000 resamples; Figure W3). Central to our theorizing, we observed a significant interaction between authoritarianism and volitional wrongdoing (β = .196, SE = .076, CI_{95}: .048, .344, t(1172) = 2.60, p = .009), such that participants higher in authoritarianism were more likely to express negative moral judgments when the professor's wrongdoing was volitional. Negative moral judgments, in turn, positively predicted willingness to make a retributive donation (β = .365, SE = .026, CI_{95}: .314, .416, t(1173) = 14.07, p < .001; index of moderated mediation: β = .072, SE = .029, CI_{95}: .016, .128).

Importantly, we observed no significant interactions between moral identity (β = .108, SE = .102, CI_{95}: -.093, .309, t(1172) = 1.06, p = .292) or reactance (β = -.100, SE = .091, CI_{95}: -.279, .079, t(1172) = -1.10, p = .274) and our experimental manipulation. Given that the interaction emerges among retribution-oriented consumers, and not those high in

moral identity or trait reactance, we find that a retribution account better accounts for the patterns observed in our data.

Figure W3: Supplementary Study 3 Results

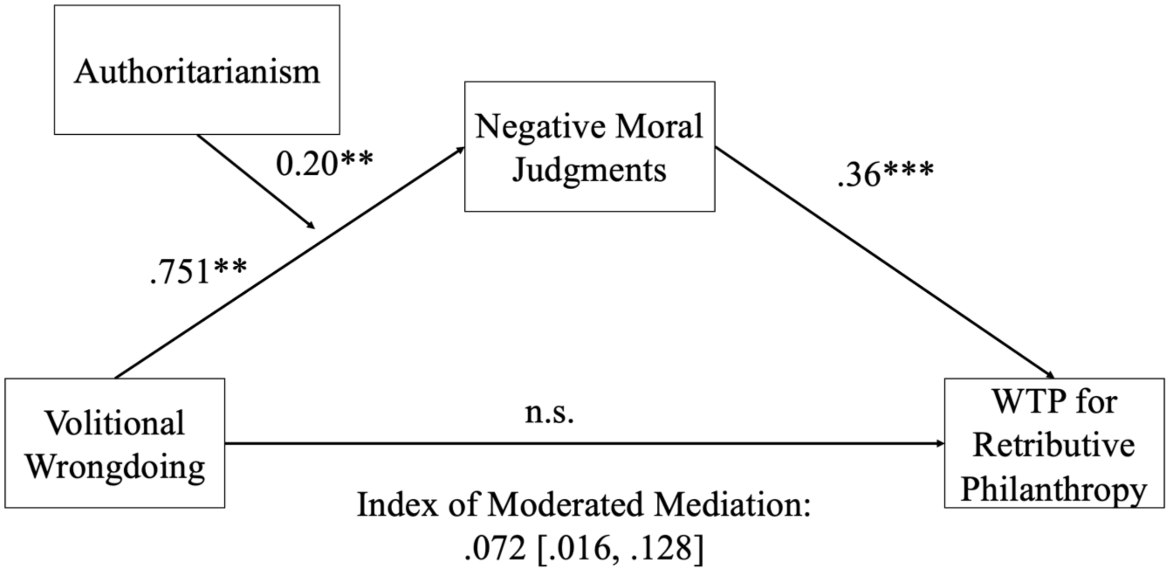

Discussion

The results of this study support the findings of Study 6 that authoritarianism, an individual-difference personality trait typically associated with retribution and punishment, is conducive to retributive donations, insofar as individuals higher in authoritarianism tend to form stronger negative moral judgments in response to volitional wrongdoing. Additionally, these results rule out two plausible alternative explanations of our proposed effects: reactance and moral identity. Taken together, these results support our conceptualization of retributive philanthropy as a retributive behavior.

Web Appendix K: Supplementary Study 4 (Study 5 Reactance and Moral Identity Follow-up Study)

The purpose of this study is to replicate the findings of Supplementary Study 3 using an alternate measure of willingness to make a retributive donation. Supplementary Study 3 used a 6-point scale with a retributive and non-retributive organization as anchors in order to match the choice-based design of Study 3 more closely. However, other studies such as Study 4 use a 7-point scale that measures likelihood of making a retributive donation, with no comparison to non-retributive organizations. In this study, we replicate the basic findings of Supplementary Study 3 with respect to the interaction between moral identity, reactance, and volitional wrongdoing but with a 7-point likelihood scale more similar to Study 4.

Method

Seven hundred ninety-two undergraduate participants (M_{age}=19.02, 52.65% female, 45.83% male, 1.51% nonbinary) from a large North American university completed this study in exchange for course credit and were randomly assigned to conditions in a two-factor (wrongdoing: volitional vs. non-volitional) between-participants design. Our data collection and analysis plans were preregistered at https://aspredicted.org/5szs-3xz7.pdf.

Participants first completed Hong and Page's (1989) Psychological Reactance scale and Aquino and Reed's (2003) Moral Identity scale. Next, they were exposed to the same stimuli used in Study 4: participants read an article describing Kanye West’s antisemitic statements, wherein West’s statements were either described as arising out of mental illness

(non-volitional) or unrelated to mental illness (volitional). Participants then completed the same measures of negative moral judgements and likelihood of making a retributive donation as used in Study 4.

Results

We first replicated our focal mediation model using Hayes' (2013) PROCESS Model 4 with bootstrap resampling. Specifically, we found that our experimental manipulation resulted in increased negative moral judgements (β = 0.292, SE = .084, CI_{95}: .128, .456, t(790) = 3.49, $p < .001$), which in turn led to higher subsequent likelihood of making a retributive donation (β = 0.474, SE = .052, CI_{95}: .371, .576, t(789) = 9.10, $p < .001$). The effect of volitional wrongdoing on likelihood of making a retributive donation was fully mediated by negative moral judgments (β = 0.138, SE = .043, CI_{95}: .059, .228).

Results were then analyzed, per our pre-registration, with two complementary moderated mediation analyses using Hayes' (2013) PROCESS Model 7 with bootstrap resampling (N=10,000), testing the moderating effects of both reactance and moral identity on the effect of volitional wrongdoing on negative moral judgments. Consistent with Supplemental Study 3, we observed no significant interactions between either reactance (β = -.079, SE = .108, CI_{95}: -.291, .132, t(788) = -0.74, p = .462) or moral identity (β = 0.017, SE = .114, CI_{95}: -.206, .239, t(788) = 0.15, p = .884) and our experimental manipulation, such that these two individual differences did not explain our observed effect of volitional wrongdoing on retributive donation. Our results are visualized in Figures W4 and W5.

Figure W4: Supplementary Study 4 Reactance Results

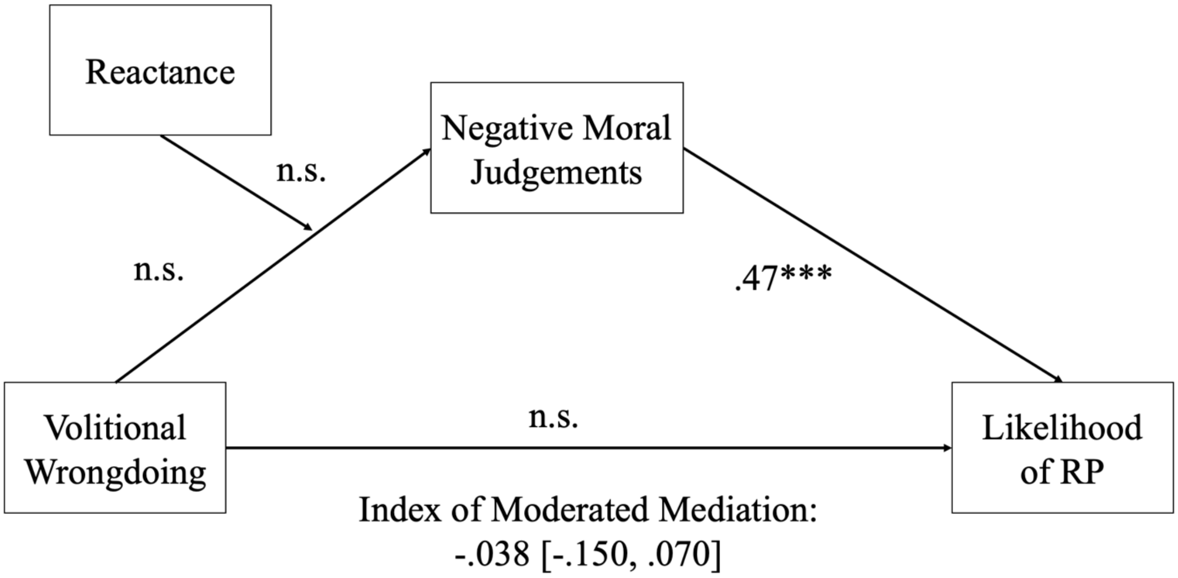

Figure W5: Supplementary Study 4 Moral Identity Results

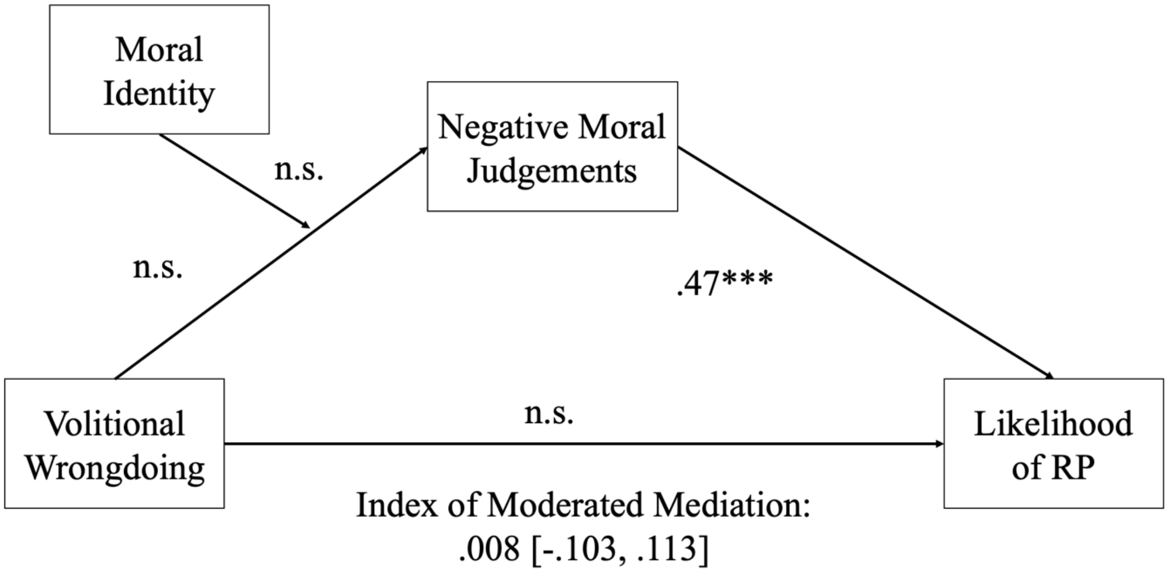

Discussion

This study replicates the findings of Study 6 using an alternate dependent measure. Specifically, this study shows that reactance and moral identity do not explain our observed effect of volitional wrongdoing on retributive donation. Additionally, this study replicates the findings of Studies 4, 5, and 7, which all identify negative moral judgments as a mechanism by which volitional wrongdoing (versus non-volitional) results in increased retributive donation behaviors.

Web Appendix L: Supplementary Study 5 (Retributive Donation vs. For-Profit Payment for Retribution)

Across our primary and supplementary studies, thus far, we have established that retributive philanthropy is distinct in many respects from traditional accounts of prosocial behavior. This supplementary study distinguishes retributive philanthropy from other accounts of retributive behavior by exploring how the charitable component of retributive philanthropy impacts consumers' willingness to pay using yet another contemporary social issue as its context.

In general, most consumers do not wish to harm others without cause (Pundak et al. 2021); however, with retributive philanthropy, we have identified a context in which consumers are interested in punishment and, arguably, find this punishment more enticing because of its pairing with charity. Thus, our final study compares retributive campaigns launched by non-profit organizations to equivalent campaigns by for-profits. We, specifically, expect that retributive donations will be perceived as less socially, legally, and professionally risky than other forms of retribution, and thus be more attractive. Whereas our previous studies highlighted how retributive philanthropy is distinct from other types of prosocial behavior, this supplementary study enables us to demonstrate that retributive philanthropy is also theoretically distinct from other types of retributive behavior–further highlighting the conceptual uniqueness of this novel phenomenon.

Method

Undergraduates (N = 598 M_{age} = 19.05, 53.67% female) from a large North American university participated for course credit. One participant was excluded for failing to complete all measures. Participants were randomly assigned to one of two conditions (Retributive Option: for-profit retributive payment vs. non-profit retributive donation) in a between-participants design. Our data collection and analysis plan are pre-registered at https://aspredicted.org/8qc4-zky6.pdf.

All participants read a contemporary news article about the Russian invasion of Ukraine, which detailed the devastating impact of the war on the Ukrainian people. To confirm that participants regarded the Russian invasion as wrong, we asked them to rate their negative moral judgment of Russia (e.g., "I believe the Russian military is wrongfully attacking Ukraine"; 3-items, 1—7; 1="Strongly disagree", 7="Strongly agree"). Next, participants learned about an organization that inscribes custom messages on weaponry used to attack the Russian military. Participants either read about 1) Ukrainian charities offering this opportunity for a $10 donation (retributive donation), or 2) a company offering this opportunity for a $10 payment (for-profit retributive payment). To measure perceived personal risk, participants then rated the extent to which the retribution option they viewed would be legally, socially, or professionally risky (e.g., "purchasing this service would be socially risky"; 1—7, 1="Strongly disagree", 7="Strongly agree"). Then, participants rated their likelihood of donating or paying for this opportunity (1—7; 1="Extremely unlikely", 7="Extremely likely").

Finally, we gave participants the opportunity to actually provide a personalized message on the Ukrainian weaponry destined for a Russian target (i.e., a soldier). We predicted that participants would be significantly more willing to inscribe vengeful messages on bullets meant for Russian soldiers when combined with donation versus a for-profit payment. Additional information on stimuli and measures is reported in Web Appendix C.

Results

First, participants reported strong negative moral judgments of the Russian invasion that were significantly above the scale midpoint (M_{NMJ} = 5.43 CI_{95}: 5.36, 5.51, t(596) = 37.57, *p* < .001), and many elected to inscribe retributive messages on the Ukrainian weaponry, including: “Bombs away”, “You started this, we are finishing it”, and “F%$# You”. These messages indicate that some participants were using the weapon inscription service to leave retributive messages. In line with our predictions, we found a positive main effect of our manipulation, such that participants were significantly more likely to inscribe a message on a bullet meant for a Russian soldier when it came in the form of a retributive donation ($M_{Willingness}$ = 2.36) compared to a payment to a for-profit company ($M_{Willingness}$ = 1.86; difference = 0.50, t(590.2) = 4.13, *p* < .001).

Next, we report the results of a Hayes’ PROCESS Model 4 mediation model with bootstrapped resampling (N=10,000) testing the mediating effect of perceived personal risk on the relationship between retribution type and willingness to pay. As expected, we found a significant effect of donation type on perceived risk, such that retributive donations were seen as less risky than other retribution (β = -.517, CI_{95}: -.705, -.329, t(595) = -5.40, *p* <

.001). Perceived risk negatively predicted willingness to pay (β = -.311, CI_{95}: -.409, -.213, $t(594) = -6.2$, $p < .001$). Overall, our index of mediation excluded zero (β = .161, SE = .043, CI_{95}: .102, .572), suggesting that perceived risk mediates the effect of retribution type on willingness to pay.

Discussion

These results demonstrate that the charitable component of retributive philanthropy increases willingness to support a retributive campaign–an effect that we observe while holding the cost and method of retribution constant. Importantly, these results also suggest that retributive philanthropy is distinct from other retributive behavior in that it is perceived as less socially, legally, and professionally risky when paired with a charitable component.

Synthesizing this study with the studies in the main manuscript, these findings establish that retributive philanthropy is not wholly prosocial or wholly retributive but, instead, is a novel form of consumer behavior that incorporates elements of both in unique ways. Managerially, these results suggest that retributive charities are advantaged relative to for-profit entities offering similar benefits for payment, and they should not forego highlighting the charitable benefit of retributive donations when making appeals to consumers.

References

Altemeyer, Bob (1996), *The Authoritarian Specter*, Cambridge, Mass: Harvard University Press.

Aquino, Karl and Americus Reed (2003), "The self-importance moral identity," *Journal of personality and social psychology*, 83, 1423–40.

Brady, William J., Killian McLoughlin, Tuan N. Doan, and Molly J. Crockett (2021), "How social learning amplifies moral outrage expression in online social networks," *Science Advances*, 7 (33), eabe5641.

Breakwell, Glynis M. and Rusi Jaspal (2022), "Coming Out, Distress and Identity Threat in Gay Men in the UK," *Sexuality Research and Social Policy*, 19 (3), 1166–77.

Brehm, Jack W. (1966), *A theory of psychological reactance*, A theory of psychological reactance, Oxford, England: Academic Press, x, 135.

Costello, Thomas H., Shauna M. Bowes, Sean T. Stevens, Irwin D. Waldman, Arber Tasimi, and Scott O. Lilienfeld (2022), "Clarifying the structure and nature of left-wing authoritarianism," *Journal of Personality and Social Psychology*, 122 (1), 135–70.

Costello, Thomas H. and Christopher J. Patrick (2021), "Development and Initial Validation of Two Brief Measures of Left-wing Authoritarianism: A Machine Learning Approach," PsyArXiv.

Grubbs, Joshua B., Brandon Warmke, Justin Tosi, A. Shanti James, and W. Keith Campbell (2019), "Moral grandstanding in public discourse: Status-seeking motives as a potential explanatory mechanism in predicting conflict," *PLOS ONE*, 14 (10), e0223749.

Haidt, J. (2001), "The emotional dog and its rational tail: a social intuitionist approach to moral judgment," *Psychological Review*, 108 (4), 814–34.

Hayes, Andrew F. (2013), *Introduction to mediation, moderation, and conditional process analysis*, Introduction to mediation, moderation, and conditional process analysis: A regression-based approach, New York, NY, US: Guilford Press, xvii, 507.

Hong, Sung-Mook and Sandra Page (1989), "A Psychological Reactance Scale: Development, Factor Structure and Reliability," *Psychological Reports*, 64 (3_suppl), 1323–26.

Karppinen, Helena, Olivia King, and Pascale Sophie Russell (2023), "Hostile emotions and close relationships: Anger can be related to constructive responses," *Personality and Individual Differences*, 212, 112258.

Lukianoff, Greg and Jonathan Haidt (2018), *The Coddling of the American Mind: How Good Intentions and Bad Ideas Are Setting Up a Generation for Failure*, New York: Penguin Press.

Lukianoff, Greg and Rikki Schlott (2023), *The Canceling of the American Mind*, New York: Simon & Schuster.

Miron, Anca M. and Jack W. Brehm (2006), "Reactance Theory - 40 Years Later," *Zeitschrift für Sozialpsychologie*, 37 (1), 9–18.

Tosi, Justin and Brandon Warmke (2016), “Moral Grandstanding,” *Philosophy and Public Affairs*, 44 (3), 197–217.

Winterich, Karen Page, K. Aquino, V. Mittal, and R. Swartz (2013), “When moral identity symbolization motivates prosocial behavior: the role of recognition and moral identity internalization.,” *The Journal of applied psychology*.
